# Heat-Stress Induced Apoptosis: A New Biotechnological Strategy to Enhance Ganoderic Acids Production in *Ganoderma lucidum*

**DOI:** 10.3390/jof12050364

**Published:** 2026-05-15

**Authors:** Meng-Hsuan Lai, Ni Tien, Hsiao-Lien Yang, Jun-He Huang, Miin-Huey Lee, Bang-Jau You

**Affiliations:** 1 Doctoral Program in Microbial Genomics, National Chung Hsing University and Academia Sinica, Taichung 402202, Taiwan; kuro8173@hotmail.com; 2 Department of Chinese Pharmaceutical Sciences and Chinese Medicine Resources, China Medical University, Taichung 404328, Taiwan; sandra19950902@gmail.com (H.-L.Y.); n0953525906@gmail.com (J.-H.H.); 3 Department of Laboratory Medicine, China Medical University Hospital, Taichung 404, Taiwan; 006719@tool.caaumed.org.tw; 4 Department of Plant Pathology, National Chung-Hsing University, Taichung 402202, Taiwan; 5 Advanced Plant and Food Crop Biotechnology Center, National Chung Hsing University, Taichung 402202, Taiwan

**Keywords:** *Ganoderma lucidum*, ganoderic acids, secondary metabolite, apoptosis, metacaspase, Bcl-2

## Abstract

*Ganoderma lucidum* is a medicinal fungus widely utilized in traditional medicine and functional foods. Its primary bioactive constituents are ganoderic acids (GAs), a group of triterpenoid compounds. While chemical-induced apoptosis has previously been shown to enhance GAs production, this study investigates the role of physical stress in this regulatory pathway. We demonstrate that heat-induced apoptosis significantly increases GAs production in *G. lucidum*. To determine whether apoptosis directly regulates this process, we overexpressed the human anti-apoptotic gene Bcl-2 in *G. lucidum*, confirming expression via RT-PCR and Western blot analysis. Upon heat-induced apoptosis, these Bcl-2 overexpression mutants exhibited increased mycelial cell viability, accompanied by reduced metacaspase activity and, notably, decreased GAs production. Furthermore, we identified a Type I metacaspase gene in *G. lucidum*, Glmca1, which contains highly conserved catalytic domains common across fungal species. Silencing of Glmca1 followed by heat-induced apoptosis led to results similar to Bcl-2 overexpression: enhanced cell viability, suppressed metacaspase activity, and a significant reduction in GAs yield. These findings provide compelling evidence that apoptosis functions as a critical regulatory mechanism for secondary metabolite production in *G. lucidum*. Consequently, modulating apoptotic pathways through physical induction offers a promising strategy for enhancing the production of bioactive ingredients in medicinal fungi.

## 1. Introduction

*Ganoderma lucidum* is an important traditional Chinese medicine (TCM) and functional food that has been widely used in Asia for thousands of years. Ganoderic acids (GAs), a group of triterpenoid compounds, are among the major bioactive constituents of *G. lucidum.* Previous studies have demonstrated that GAs exhibit diverse pharmacological activities, including antiviral and antitumor effects [1,2,3]. In addition to its clinical applications in TCM, *G. lucidum* is also extensively used as a functional food ingredient. Therefore, developing effective strategies to enhance GAs production is of significant importance for both research and industrial applications.

The mechanisms underlying apoptosis have been extensively characterized in mammals; however, apoptotic processes in fungi and plants remain comparatively less well understood. Programmed cell death (PCD) is essential for organismal development and homeostasis, playing critical roles in processes such as tissue elimination and organ morphogenesis [4]. In mammals, apoptosis is mediated through two major pathways: the extrinsic and intrinsic pathways. The extrinsic pathway is initiated by death receptor signaling, whereas the intrinsic pathway is governed by mitochondrial regulation.

Members of the Bcl-2 (B-cell lymphoma 2) protein family are central regulators of the intrinsic apoptotic pathway in mammals, worms, and insects [5,6]. Anti-apoptotic Bcl-2 proteins counteract the activity of pro-apoptotic factors such as Bax and Bak, whose activation increases mitochondrial outer membrane permeability. This permeabilization facilitates the release of apoptogenic factors, such as cytochrome *c*, thereby triggering caspase activation and the execution of apoptosis [7].

Apoptosis has been documented in both yeast and filamentous fungi [8,9,10], where it regulates a broad range of physiological processes, including aging, pathogenesis, and heterokaryon incompatibility [11]. Fungal apoptosis can be triggered by diverse environmental stresses, such as oxidative and fungicidal stress in *Aspergillus fumigatus* [12], heavy metal exposure in aquatic fungi [13], osmotic stress in *Fusarium proliferatum* [14], and heat stress in *Pleurotus* species [15]. In addition to environmental cues, genetic perturbations can also induce programmed cell death; for example, heterologous expression of apoptotic genes has been shown to trigger apoptosis in *Colletotrichum gloeosporioides* [16].

However, the mechanisms underlying fungal apoptosis, particularly in the division *Basidiomycota*, have not yet been clearly elucidated. Several homologs of mammalian apoptosis-related genes have been identified in fungi, including *Yca1*, a metacaspase and functional ortholog of mammalian caspases, and *Nma111*, a nuclear mediator of apoptosis, in *Saccharomyces cerevisiae* [17,18]. These fungal homologs of mammalian apoptosis genes exhibit similar functional roles in mediating programmed cell death under stress conditions.

Although fungi lack endogenous homologs of Bcl-2 [11], studies have shown that heterologous expression of Bax in *S. cerevisiae* induces apoptosis, and that Bcl-2 can inhibit Bax-mediated cell death [19]. Moreover, overexpression of human Bcl-2 in *C. gloeosporioides* suppresses apoptosis and enhances fungal virulence [16]. These findings suggest that mammalian Bcl-2 family proteins can modulate apoptotic processes in fungi and indicate the existence of conserved regulatory mechanisms governing programmed cell death in fungi and mammals.

Fungal metacaspases are classified as Type I metacaspases [20] and were originally identified in yeast, where they mediate many apoptotic processes. Most fungi typically possess one or two metacaspase genes [21]. These genes play important roles in programmed cell death [18], development [22], aging [23], sporulation [11], and pathogenicity [24]. Previous studies have shown that hydrogen peroxide (H_2_O_2_) induces apoptosis in *A. fumigatus* and *S. cerevisiae* [25]. In addition, disruption of the yeast metacaspase gene *YCA1* inhibits apoptosis and enhances resistance to oxidative stress [18]. Various environmental stressors have been reported to trigger apoptosis in fungi, often accompanied by increased metacaspase activity. For example, acetic acid induces an apoptotic response in *S. cerevisiae* characterized by a significant increase in metacaspase activity [26]. Similarly, heat stress induces apoptosis in the filamentous fungus *Rhizopus oryzae*, along with a concomitant increase in metacaspase activity [27].

The role of apoptosis in regulating diverse physiological processes has been extensively investigated across multiple organisms. However, it remains unclear whether apoptosis broadly regulates secondary metabolite production in plants and fungi. In *Artemisia annua*, an annual herbaceous plant, oligosaccharides extracted from the endophytic fungus *Colletotrichum* sp. were shown to induce apoptosis in hairy roots and enhance artemisinin biosynthesis [28]. Similarly, crude extracts of *Fusarium oxysporum* trigger apoptosis in suspension cultures and increase the production of the anticancer compound taxol in *Taxus chinensis* [29]. Zhu et al. further demonstrated that silencing the transcription factor *PacC* in *G. lucidum* leads to apoptosis-associated DNA fragmentation and reactive oxygen species (ROS) accumulation, accompanied by increased GAs biosynthesis [30]. Our previous work has also shown that chemical inducers, such as aspirin and cyclic adenosine monophosphate (cAMP), can induce apoptosis and promote GAs production in *G. lucidum* [31,32]. Despite these advances, the necessity of removing chemical or biological inducers during downstream processing represents a significant bottleneck for industrial-scale production.

In this study, we demonstrate that physical heat stress acts as a non-invasive stimulus that promotes GAs production by inducing apoptosis. We further investigated whether this apoptotic pathway directly regulates secondary metabolite synthesis in *G. lucidum.* For this purpose, we generated mutants overexpressing the human Bcl-2 protein as well as strains with silenced metacaspase genes. Following heat treatment, overexpression of *Bcl-2* and silencing of metacaspase genes reduced apoptosis and, consequently, suppressed GAs production. These results indicate that apoptosis directly modulates the production of secondary metabolites in *G. lucidum*, revealing a mechanistic link between programmed cell death and GAs production.

## 2. Materials and Methods

### 2.1. Fungal Strain and Culture Condition

The basidiomycete fungus *G. lucidum* strain BCRC 36111, obtained from the Bioresource Collection and Research Center (BCRC) at the Food Industry Research and Development Institute (Hsinchu, Taiwan), was used in this study. For routine experiments, the strain was maintained by transferring a fresh mycelial agar disk to the center of a Potato Dextrose Agar (PDA; Becton Dickinson, Franklin Lakes, NJ, USA) plate. Mycelia were cultured on PDA for 5–7 days and subsequently harvested for genomic DNA, RNA, and protein extractions.

### 2.2. Identification and Structural Analysis of Glmca1 Gene in G. lucidum

We identified the conserved metacaspase sequence (ANMIRAMQWLVKDAQPNDSLFLHYSGHGGQT) from a previous study [33] and used it as a query in a tBLASTn search against the *G. lucidum* strain BCRC 36111 genome database (JAAIFM000000000.1). A putative gene candidate, designated *Glmca1*, was identified. The cDNA of *Glmca1* was cloned and sequenced. The obtained cDNA sequence was analyzed using the Gene Structure Display Server 2.0 (GSDS; https://gsds.gao-lab.org/) to identify introns and exons. The encoded protein was further characterized using InterProSan version 108.0 (European Bioinformatics Institute, EMBL-EBI, Hinxton, UK; https://www.ebi.ac.uk/interpro/), and the NCBI Conserved Domain Search Service (CD-Search).

Type I and Type II metacaspases from plants [34,35,36] and fungi (Table S1) were used for Glmca1 phylogenetic analysis. Multiple sequence alignment was performed using the MUSCLE algorithm integrated in MEGA X (version 10.x; Penn State University, Centre County, PA, USA). Phylogenetic trees were constructed using the Neighbor-Joining (NJ) method, and evolutionary distances were calculated using the JTT (Jones–Taylor–Thornton) model. The reliability of the tree topology was assessed by bootstrap analysis with 1000 replicates.

### 2.3. Plasmids and Construction

In this study, the binary vector pCAMBIA0380 was used for the overexpression of human Bcl-2 and for silencing the metacaspase gene in *G. lucidum*. To achieve high-level expression of Bcl-2, the human *Bcl-2* gene (GenBank: NP_000624.2) was codon-optimized for *G. lucidum* and placed under the control of the glyceraldehyde-3-phosphate dehydrogenase (GPD) promoter (GenBank: DQ404345.1) and the *Aspergillus nidulans* trpC terminator (GenBank: X02390.1) within pCAMBIA0380.

To silence the metacaspase gene in *G. lucidum*, a dual promoter system comprising the GPD and 35S promoters was used to express sense and antisense RNA for gene-silencing. The orotidine 5′-monophosphate decarboxylase (*URA3*) gene has been employed as a selection marker in previous studies [37]. The *URA3* sequence was amplified from the *G. lucidum* BCRC 36111 genome using a primer set P1 designed based on *G. lucidum* G20 strain (GenBank: AFR33965.1). An 812 bp fragment of the *URA3* coding region (Supplementary Materials S1) was used both as a gene silencing control and as a selection marker. The dual promoters driving the 836 bp *URA3* fragment were synthesized by AllBio Science Incorporated, and a multiple cloning site (MCS) was incorporated within the construct in the following configuration: GPD promoter–MCS–URA3–35S promoter. This silencing construct was subsequently cloned into pCAMBIA0380 to generate pURA3-dual promoter.

For the metacaspase gene-silencing construct, an 850 bp fragment of metacaspase cDNA (Supplementary Materials S2) was amplified from *G. lucidum* BCRC 36111 using the primer pair P2, followed by nested PCR with primer P3. The resulting 850 bp fragment was cloned into the multiple cloning site (MCS) of pURA3-dual promoter using XmaI and SpeI restriction sites. To confirm that URA3 silencing did not affect the functional analysis of metacaspase, the metacaspase fragment was replaced with the *eGFP* sequence. The *eGFP* gene (GenBank: AAT34982.1) was amplified from pCT74 using primers P4 and subsequently cloned into pURA3-dual promoter via XmaI and SpeI.

All primers used in this study are listed in Table S2.

### 2.4. Agrobacterium Transformation and Fungal Protoplast Preparation

*Agrobacterium tumefaciens* strain AGL-1 was first streaked on LB agar (LA) plates supplemented with 100 μg/mL kanamycin and incubated at 28 °C for 3 days. A single colony was then inoculated into LB liquid medium containing 100 μg/mL kanamycin and grown at 28 °C with shaking for 19 h. The culture was subsequently diluted to an optical density at 550 nm (OD_550_) of 0.25 in induction medium (IMM) containing acetosyringone (AS) and incubated at 28 °C with shaking at 250 rpm for 5 h. The prepared bacterial cells were then used for fungal transformation following the protocol described in Shi et al. [38].

To prepare *G. lucidum* mycelia for *Agrobacterium tumefaciens*-mediated transformation, the fungus was first grown on PDA plates for 7 days. Approximately 0.85 g of mycelia were collected and cultured in 200 mL of CYM liquid medium at 28 °C with shaking at 130 rpm for 3 days. The mycelia were then harvested by centrifugation at 4900 rpm for 10 min, homogenized, and further cultured in 200 mL of fresh CYM liquid medium at 28 °C with shaking at 140 rpm for 20 h. The mycelia were washed twice with 0.6 M mannitol by centrifugation at 12,000× *g* for 10 min and subsequently digested with 2% lysing enzyme (Sigma, St. Louis, MO, USA) at a volume 2.5 times the mycelial wet weight for 2–3 h. After digestion, the mixture was washed twice with 0.6 M mannitol by centrifugation at 3500× *g* for 10 min. Finally, the mycelial pellet was mixed with the AGL-1 suspension at a 1:1 (*v*/*v*) ratio and spread onto 0.45 μm membrane filters (Whatman, Maidstone, UK) for transformation.

### 2.5. Screening of Transformants by Selection Media

After co-culturing at 25 °C for 4 days, the membranes were transferred onto PDA plates containing 40 μg/mL hygromycin B and 200 μg/mL cefotaxime. The membranes were removed after 3–4 days, and individual colonies were picked and subcultured on PDA plates supplemented with 50 μg/mL hygromycin B at 28 °C for 3 days. The resulting transformants were then screened for high Bcl-2 expression or effective metacaspase gene silencing.

To identify transformants with high Bcl-2 expression, mycelial agar plugs from 3-day-old colonies grown on 50 μg/mL hygromycin B were transferred to medium containing 1 mg/mL hygromycin B. After 3–7 days, colonies with diameters ≥ 1 cm were selected. These candidate transformants were subcultured three times before being preserved for subsequent experiments.

To identify potential metacaspase-silencing transformants, mycelial agar plugs from 3-day-old colonies were transferred to medium containing 200 μg/mL 5-fluoroorotic acid (5-FOA; Thermo, Waltham, MA, USA). After 3 days, colonies exhibiting notably rapid growth were selected and subcultured three times before being preserved for further analysis.

To detect the presence of the transgenes, genomic DNA was extracted from peripheral mycelia after 6 days of growth, as previously described [39]. For *Bcl-2* overexpression transformants, DNA sequences corresponding to the GPD promoter, *Bcl-2*, and the terminator were amplified by PCR using primer pairs P5, P6, and P7. The hygromycin resistance gene was amplified using primer pair P8. For *Glmca1*-silencing transformants, PCR assays were performed using primer pairs P10, P11, and P12. For URA3-silencing transformants, primer pairs P10_F and P12_R were used to assess the presence of the transgene.

To analyze gene expression, total RNA was extracted from 6-day-old mycelia using TRIzol reagent. The RNA samples were treated with DNase I (Ambion™, Thermo Fisher Scientific, Waltham, MA, USA; Cat. No. AM2222) at 37 °C for 30 min to remove contaminating genomic DNA. Reverse transcription was then performed using oligo-dT primers and SuperScript III (Invitrogen™, Thermo Fisher Scientific, Waltham, MA, USA; Cat. No. 18080051). Semi-quantitative RT-PCR was used to detect transcripts of *Bcl-2*, *Glmca1*, and *URA3*. The *G. lucidum GPD* gene served as an internal control and was amplified using primer pair P9. For *Bcl-2* expression, primer pair P6 was used. To assess silencing of *URA3* and *Glmca1*, primer pairs P13–P15 and P16_F/P2_R were used, respectively.

### 2.6. Western Blotting

Mycelia from wild type and *Bcl-2* transformants were collected for protein extraction. The mycelia were ground into a fine powder using liquid nitrogen, and 200 mg of the sample was resuspended in 500 μL lysis buffer (50% glycerol, 20 mM Tris-HCl, pH 7.5, 1 mM EDTA, 0.1 mM DTT, and 0.2 mM PMSF). The mixture was centrifuged at 13,500 rpm at 4 °C, and 300 μL of the resulting supernatant was mixed with 100 μL of sample loading dye. Samples were boiled for 10 min and subjected to Western blotting. A total of 50 μg of total protein was separated on a 10% SDS-PAGE gel. Bcl-2 protein was detected using Bcl-2 (D55G8) Rabbit mAb (Human specific; Cell Signaling Technology, Danvers, MA, USA; Cat. No. 4223), while β-actin served as a loading control and was detected using anti–β-actin antibody (Abcam, Waltham, MA, USA; Cat. No. Ab8224).

### 2.7. Phenotypic Characterization and Growth Analysis

A fresh mycelial agar disk was placed at the center of a 9 cm PDA plate and incubated at 28 °C for 6 days, with colony diameters measured daily. Each strain was tested in triplicate (n = 3), and the resulting data were analyzed statistically. Phenotypic morphology was photographed and recorded on day 5 of incubation.

### 2.8. Heat Treatment

Mycelia used for heat treatment were collected from 7-day-old PDA cultures and fragmented by placing them in a 1.5 mL microtube and grinding with a pellet pestle. Approximately 50 mg of mycelial fragments were spread onto cellophane overlays on PDA plates and incubated at 28 °C for 5 days. For Evans blue staining, Annexin V analysis, and caspase activity assays, 0.2 × 0.2 cm^2^ mycelial cellophane disks were excised and transferred to a Petri dish containing 25 mL PDB, followed by incubation at 28 °C for 15 h. Once fresh mycelia had emerged (~12 h), the mycelial disks were subjected to heat treatment. Three disks were placed in a 5 cm Petri dish containing 15 mL PDB and incubated at 43 °C or 45 °C for heat treatment, or at 28 °C as a control, with shaking at 100 rpm. After a 1 h recovery period at 28 °C, the disks were used for apoptosis-related assays.

For GAs production assays, mycelial cellophanes were transferred to flasks containing 25 mL PDB and incubated under the same heat treatment conditions or control (28 °C) with shaking at 100 rpm. To enhance GAs production, the mycelia from both control and heat-treated groups were further incubated at 32 °C for 12 h.

### 2.9. Assay of Metacaspase Activity

Metacaspase activity was assessed using the CaspACE™ FITC-VAD-FMK In Situ Marker (Promega, Madison, WI, USA; Cat. No. G7461) following the method described by [27]. After heat treatments, mycelia were washed twice with PDB and incubated with 10 μM FITC-VAD-FMK at 28 °C for 1 h in the dark. Fluorescence was observed using a fluorescence microscope with an excitation wavelength of 495 nm, and images were captured immediately using a CCD camera (IX70, Olympus, Tokyo, Japan) under identical settings for all samples.

Caspase activity was quantified using ImageJ software (https://imagej.net/ij/, National Institutes of Health, Bethesda, MD, USA) by calculating the ratio of green fluorescent mycelial area to the total mycelial area within a single field of view under a 40× objective lens. For each strain, three mycelial pieces were prepared, and 4–6 fields of view were recorded per piece. From these, eight representative fields of view per strain were selected for fluorescence quantification and statistical analysis.

### 2.10. Analysis of Phosphatidylserine Externalization by Annexin V Staining

Phosphatidylserine (PS) externalization was assessed using the FITC Annexin V Apoptosis Detection Kit I (BD Biosciences, San Jose, CA, USA). After heat treatments, mycelial cellophane disks were incubated with 2% lysing enzyme for 30 min to degrade the cell wall. Following digestion, the mycelia were washed twice with PBS and then incubated with FITC Annexin V and propidium iodide (PI) for 15 min at room temperature in the dark. Fluorescence was observed using a fluorescence microscope with an excitation wavelength of 488 nm.

### 2.11. Cell Viability Assay via Evans Blue Staining

Evans blue staining was used to quantify dead cells [40]. After heat treatments, samples were stained with 1% Evans blue for 10 min and then washed with 1× PBS. The Evans blue density was calculated as the ratio of clearly stained mycelia to the total number of mycelia within a single field of view under a 40× objective lens. For each strain, three mycelial samples were prepared, with 6–8 fields of view recorded per sample. From these, 6–10 fields of view per strain were randomly selected for quantification and statistical analysis, with each field containing 50–100 identifiable mycelial filaments.

### 2.12. Ganoderic Acids Production Analysis

After heat treatments, mycelia were collected, pressed dry with paper towels, and dehydrated in an oven for 2–4 days. The dried mycelia were weighed and ground into a fine powder. For GAs extraction, 50 mg of the powdered sample was frozen in liquid nitrogen and pulverized. The sample was then suspended in 3 mL of methanol and extracted on a shaker at 125 rpm for 18 h, followed by ultrasonic extraction for 30 min and a subsequent 2 h shaking extraction at 125 rpm. The mixture was centrifuged at 3900 rpm for 15 min, and the supernatant was collected and filtered through a 0.22 μm membrane filter. Total GA content was quantified by High-Performance Liquid Chromatography (HPLC) using 10 μL of extract per injection. Briefly, the HPLC analysis followed the method described by Chyr and Shiao, 1991 [41], where nearly all identified peaks correspond to triterpenoids. Consistent with our previous studies [31,32,42], the total GA content was calculated by integrating all peak areas eluted between 5 and 50 min. The total amount was then quantified using a GA24 standard, identified in our previous work [42], to convert peak areas into micrograms (μg).

### 2.13. Statistical Analysis

All quantitative data are presented as mean ± SEM (standard deviation of the mean) from at least three independent experiments (n = 3). Comparisons between two experimental groups were performed using a two-tailed, unpaired Student’s *t*-test. A *p*-value < 0.05 was considered statistically significant. Specifically, * *p* < 0.05, ** *p* < 0.01, and *** *p* < 0.001 indicate different levels of statistical significance.

## 3. Results

### 3.1. Heat-Induced Cell Apoptosis and GAs Production in G. lucidum

**Figure 1:**
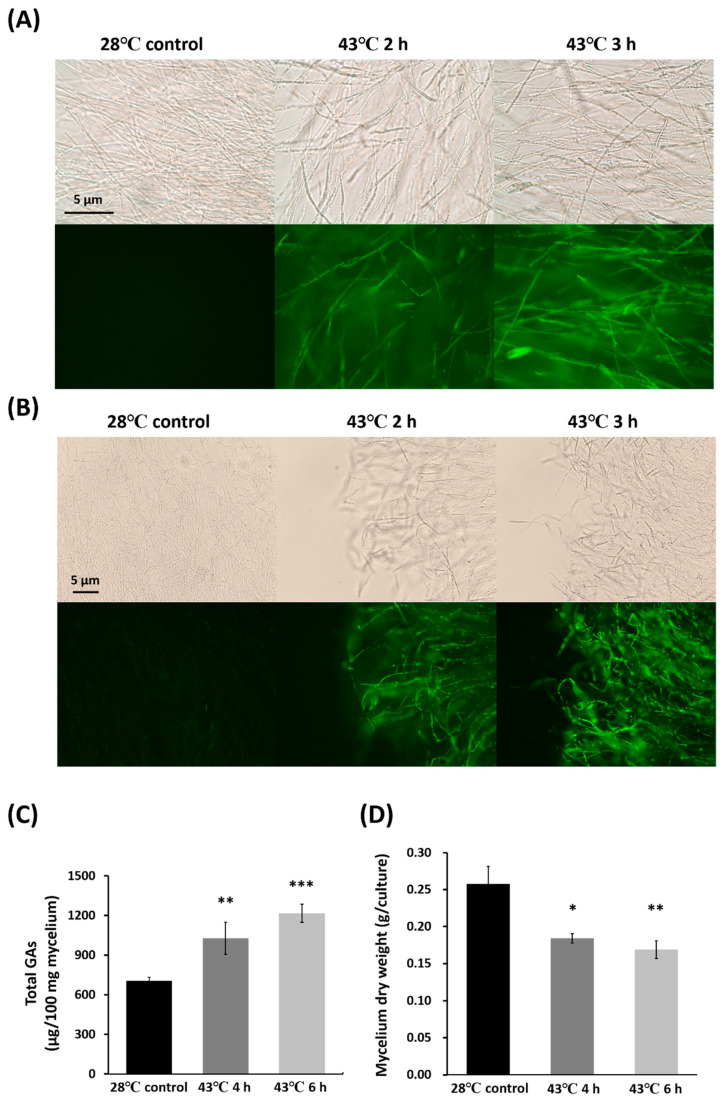
Heat-induced apoptosis and ganoderic acids (GAs) production in *Ganoderma lucidum*. Mycelia were subjected to heat treatment at 43 °C for 2 or 3 h. Caspase activity was detected using the CaspACE™ FITC-VAD-FMK In Situ Marker (**A**), and phosphatidylserine (PS) externalization was analyzed by Annexin V staining (**B**). More than ten independent samples were analyzed, and representative images are shown. GAs production (**C**) and mycelial dry weight (**D**) were measured after heat treatment at 43 °C for 4 or 6 h. Values represent the mean ± SD (n = 3). Statistical significance is indicated as * *p* < 0.05, ** *p* < 0.01, *** *p* < 0.001 compared with the control.

Heat treatment was used to induce apoptosis in *G. lucidum*. We first verified whether heat effectively triggers apoptosis and enhances GAs production. Activated metacaspase and phosphatidylserine externalization are commonly used markers for detecting apoptosis. Mycelia were exposed to 43 °C for 2–3 h and 45 °C for 3 h, after which metacaspase activity was assessed using the CaspACE™ FITC-VAD-FMK In Situ Marker. An increase in metacaspase activity, indicated by green fluorescence, was observed as early as 2 h post-treatment compared to the 28 °C control. Metacaspase activity further increased at 3 h under heat treatment relative to the 2 h time point (Figure 1A). Consistent with the results obtained at 43 °C for 2–3 h, caspase activity was also significantly detected in mycelia treated at 45 °C for 3 h (Figure S1A).

Furthermore, Annexin V staining was used to assess phosphatidylserine externalization. The results showed that mycelia exhibited phosphatidylserine externalization after 2 h of heat treatment at 43 °C, and the extent of externalization increased in a time-dependent manner with longer exposure (Figure 1B). To further understand the progression of cell death, propidium iodide (PI) staining was performed. As shown in Figure S1B, weak PI signals were observed at 2 h under 43 °C, which increased at 3 h post-treatment. Notably, both PI and Annexin V signals were strong at 3 h under 43 °C. Together, these results indicate that heat treatments at 43 °C and 45 °C primarily induce apoptosis in *G. lucidum*. The intensity of PI staining was much stronger at 45 °C than at 43 °C, indicating increased late apoptosis at the higher temperature. Therefore, 43 °C heat treatment was used for further investigation.

We next investigated whether heat-induced apoptosis affects GAs production. The results showed that GAs levels increased significantly after treatment at 43 °C for 4 or 6 h (Figure 1C), with increases of 45.9% and 72.3%, respectively. Under heat stress, mycelial dry weight decreased significantly compared to the control (Figure 1D), with reductions of 28.4% and 34.3% for the respective treatments. Together, these results demonstrate that heat treatment promotes apoptosis and enhances GAs production in *G. lucidum*. In a separate experiment, we treated the mycelia at 28, 35, 40, and 45 °C for 2 days and assessed GAs production and fungal dry weight. We found that GAs production significantly increased (Figure S2A), while dry weight was significantly reduced at both 40 and 45 °C (Figure S2B). However, the 35 °C treatment did not influence GA production, despite fungal dry weight being reduced by approximately 10% compared to the control (28 °C) treatment (Figure S2A,B). Additionally, the growth of *G. lucidum* on PDA was inhibited by 39% and 63% (based on colony diameter) under 32 and 35 °C treatments after 3 days of incubation, respectively. These data suggest that heat stress below the apoptotic threshold (e.g., 35 °C) is insufficient to induce GAs production.

The HPLC profiles for GAs collected from mycelia under the control treatment (28 °C) and heat treatments (43 °C for 4 and 6 h) are shown in Figure S3A. No notable differences in peak patterns were observed between the control and heat treatments (Figure S3). However, most peaks had significantly larger areas in the heat treatment group than in the control (Figure S3C). The production of GA24 was also significantly enhanced by heat treatment (Figure S3C).

### 3.2. Bcl-2 Overexpression in G. lucidum Was Demonstrated with Protein Production

**Figure 2:**
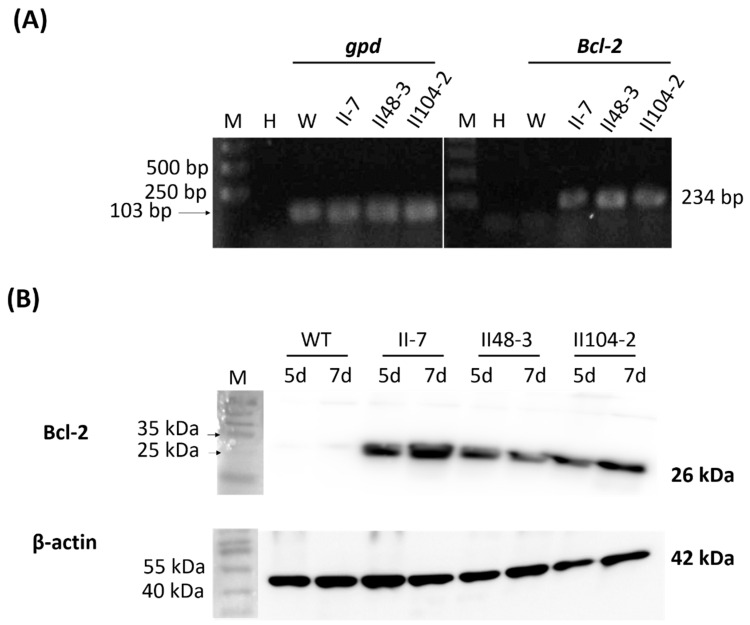
Heterologous expression of human Bcl-2 in *Ganoderma lucidum*. (**A**) *Bcl-2* expression was detected by semi-quantitative RT-PCR using RNA extracted from the wild type strain (W) and transformants (II-7, II48-3, and II104-2), with *gpd* as the internal control. M, DNA marker; H, water (PCR negative control). The PCR products for *Bcl-2* and *gpd* were 234 bp and 103 bp, respectively. (**B**) Western blot analysis of total proteins extracted from mycelia cultured on PDA for 5 or 7 days, showing human Bcl-2 protein expression (26 kDa) in the three Bcl-2 overexpression mutants. β-actin (42 kDa) was used as the loading control. The protein molecular marker (M, pre-stained protein ladder) is provided on the left of the Western blots.

The human Bcl-2 gene was introduced into *G. lucidum* via Agrobacterium-mediated transformation. Following selection on hygromycin, genomic DNA was extracted, and the presence of the transgene was confirmed by PCR (Figure S4). RT-PCR analysis revealed that Bcl-2 mRNA was expressed in transformants II7, II48-3, and II104-2 (Figure 2A). To verify protein expression, total protein was extracted from mycelia cultured for 5 or 7 days and analyzed by Western blotting. The Bcl-2 protein was readily detected in all transformants, with high levels observed in both 5- and 7-day-old mycelia (Figure 2B).

The Bcl-2 overexpressing mutants and the wild type strain were cultured on PDA, and colony diameters were measured daily for six consecutive days. The mean colony diameter and colony morphology of the mutants were not significantly different from those of the wild type strain (Figure S5). These results indicate that heterologous overexpression of Bcl-2 does not affect colony growth or morphology in *G. lucidum*.

### 3.3. Bcl-2 Overexpression Delayed Fungal Apoptosis and Enhanced Cell Viability in G. lucidum

**Figure 3:**
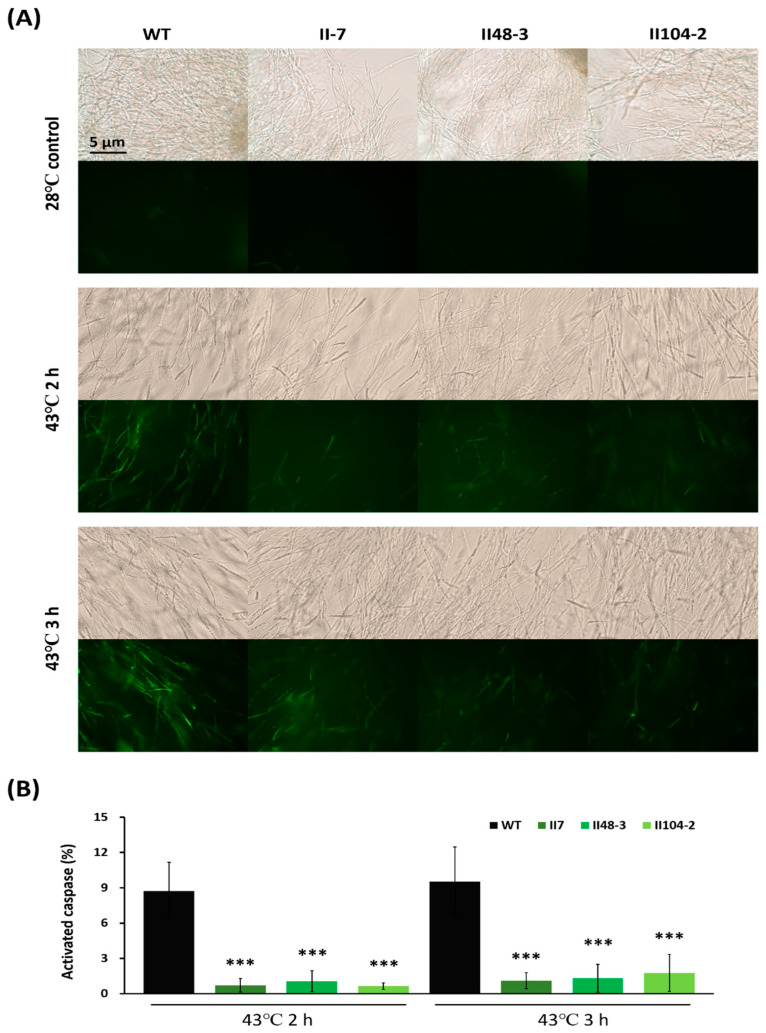
Caspase activity in *Ganoderma lucidum* Bcl-2 overexpression strains. Mycelia were subjected to heat stress at 43 °C for 2 or 3 h. (**A**) Representative images showing activated caspase-like activity in the wild type and Bcl-2 overexpression mutants post-heat treatment, detected using the CaspACE™ FITC-VAD-FMK in situ marker. (**B**) Quantification of mycelia exhibiting green fluorescence from eight independent images. Data are presented as activated caspase activity (%) relative to the 28 °C control treatment. Values are presented as mean ± SD. Statistical significance is indicated as *** *p* < 0.001 compared with the control group.

To assess whether overexpression of the human anti-apoptotic protein Bcl-2 delays apoptosis in *G. lucidum*, caspase activity was measured in mycelia. Mycelia were subjected to 43 °C for 2 or 3 h. The percentage of mycelia displaying green fluorescence was calculated. Bcl-2 overexpressing mutants exhibited significantly lower caspase activity (less than 2%) compared with the wild type (approximately 9%), indicating that Bcl-2 suppresses caspase activation in *G. lucidum*, thereby inhibiting apoptosis (Figure 3A,B).

**Figure 4:**
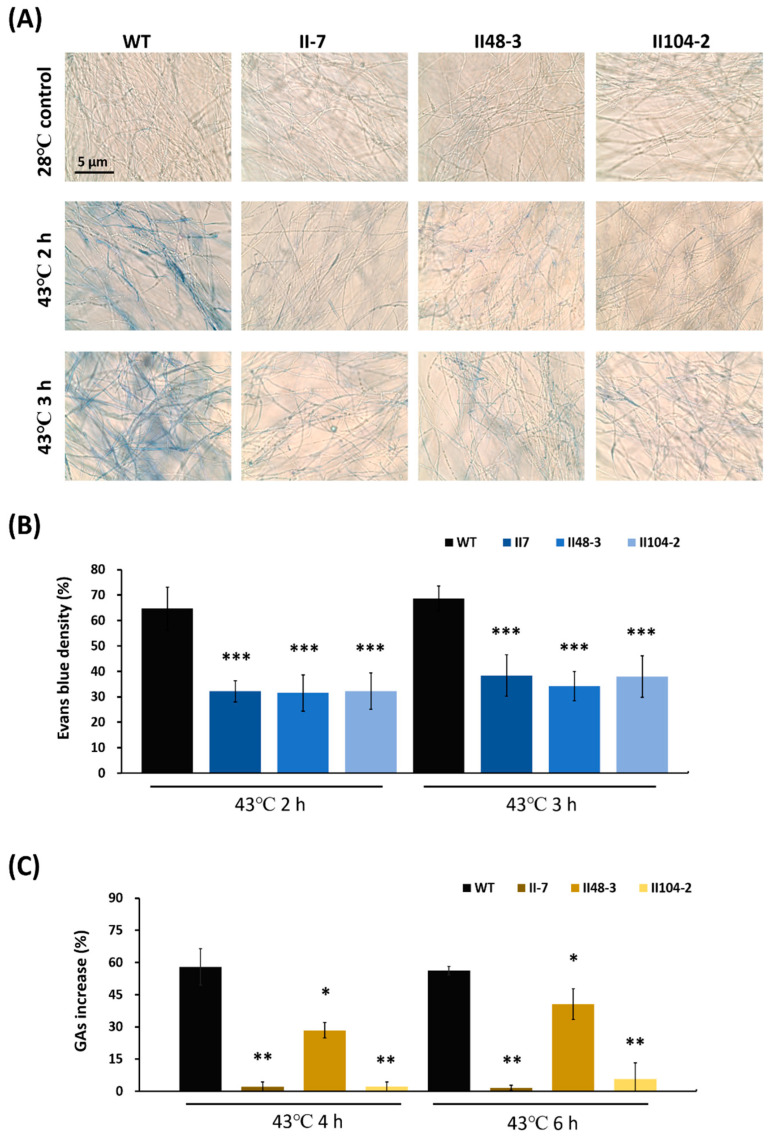
Cell viability and ganoderic acids (GAs) production in *Ganoderma lucidum Bcl-2* overexpression strains. Mycelia were subjected to heat stress at 43 °C for 2 or 3 h. (**A**) Cell viability in the wild type and Bcl-2 overexpression mutants was analyzed using Evans blue staining. (**B**) Quantification and statistical analysis of Evans blue-stained mycelia from ten independent images. Bcl-2 overexpression mutants exhibited significantly higher cell viability compared with the wild type. Data are presented as Evans blue density (%) relative to the 28 °C control treatment. (**C**) Wild type and Bcl-2 overexpression mutants were treated at 43 °C for 4 or 6 h, and GAs were quantified. Values represent the mean ± SD (n = 3). Data are presented as GAs increase (%) relative to the 28 °C control treatment. Statistical significance is indicated as * *p* < 0.05, ** *p* < 0.01, *** *p* < 0.001 compared with the control group.

Evans blue staining was performed to evaluate cell viability in the Bcl-2 overexpressing mutants following the same heat treatment. The percentage of blue-stained mycelia relative to total mycelia was determined microscopically. Compared with the wild type, which showed 64% and 68% Evans blue-positive mycelia after 2 and 3 h of heat treatment, respectively, the Bcl-2 overexpressing mutants II-7, II48-3, and II104-2 exhibited significantly higher cell viability, with Evans blue-positive values of 32% and 38%, 31% and 34%, and 32% and 37%, respectively (Figure 4A,B).

### 3.4. Bcl-2 Overexpression Reduced GAs Production During Fungal Apoptosis

We next examined whether blocking the apoptosis pathway affects GAs production. Bcl-2 overexpressing mutants II-7, II48-3, and II104-2 exhibited markedly lower increases in GAs levels compared with the wild type following 43 °C treatment. Specifically, after 4 or 6 h of heat treatment, the increases were 2.1% and 1.6% in II-7, 28.4% and 40.6% in II48-3, and 2.1% and 5.6% in II104-2, whereas the wild type showed increases of 57.9% and 56.2% (Figure 4C). These data indicate that Bcl-2 overexpression not only delays apoptosis in *G. lucidum* but also suppresses GAs production. Furthermore, under 43 °C treatment, both wild type and Bcl-2 overexpressing strains exhibited a significant reduction in mycelial biomass (Figure S6).

### 3.5. A Type I Metacaspase Gene Was Identified in G. lucidum

A cDNA of the metacaspase gene *Glmca1* from *G. lucidum* BCRC36111 was cloned and sequenced, which encodes 362 amino acids (Figure S7A). Protein domain analysis using the InterProSan and NCBI Conserved Domain Database (CDD) revealed the presence of a peptidase C14 conserved domain, classifying it within the caspase superfamily (Figure S7B). The metacaspase domain contains a P20 subunit, which carries a catalytic His-Cys dyad (Figure S7C), showing high conservation with metacaspases from yeast and other ascomycetes. In addition, 54 proline residues were found within the 157 amino acids of the N-terminus (Figure S7C).

**Figure 5:**
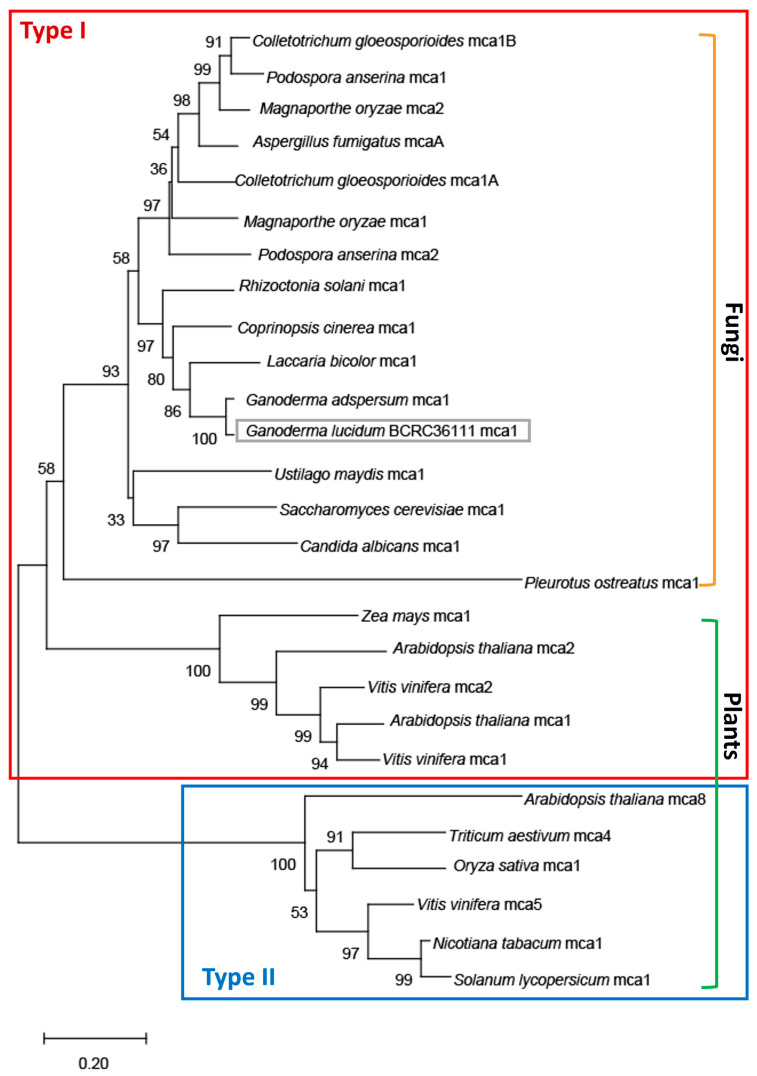
Multiple sequence alignment and phylogenetic analysis of *Ganoderma lucidum* Glmca1 and metacaspases from 12 fungal and 7 plant species. The phylogenetic tree was constructed using the Neighbor-Joining method in MEGA X. Type I and Type II metacaspases are enclosed in red and blue boxes, respectively. *G. lucidum* Glmca1 is located within the Type I metacaspase clade and is highlighted by a gray box.

Phylogenetic analysis was conducted by aligning the Glmca1 protein sequence with metacaspase homologs from 12 fungal species (including ascomycetes and basidiomycetes) and seven plant species (Table S1). Type I and Type II metacaspases were clearly separated into distinct clades. Glmca1 from *G. lucidum* clustered within the Type I metacaspase clade, closely related to other Agaricomycetes fungi, including *Laccaria* and *Coprinopsis* (Figure 5).

### 3.6. Metacaspase Gene Glmca1 Was Successfully Silenced in G. lucidum

To further investigate whether activation of apoptosis signaling regulates secondary metabolite biosynthesis, we targeted the metacaspase gene in *G. lucidum*. A co-silencing strategy was employed to reduce metacaspase expression, thereby delaying apoptosis signaling. Using Agrobacterium-mediated transformation, the binary vector pCAMBIA0380—containing dual promoters (GPD and 35S), a full-length URA3 gene, and a partial conserved metacaspase sequence—was introduced into *G. lucidum*. Putative transformants were selected on medium containing 200 μg/mL 5-FOA and subsequently verified by PCR (Figure S8). Four transformants were chosen for gene expression analysis: two carrying the URA3-silencing construct (U534 and U594) and two carrying the metacaspase–URA3-silencing construct (Y58 and Y665). Compared with the wild type, mutants Y58 and Y665 exhibited significantly reduced metacaspase expression (Figure S9). Similarly, URA3 mRNA levels were markedly decreased in the URA3-silencing mutants U534 and U594.

### 3.7. The Growth of G. lucidum Was Not Significantly Affected by Glmca1 Silencing

To assess the effects of reduced metacaspase expression on fungal growth, the strains were cultured on PDA, and colony diameters were measured daily. The growth of all silencing mutants was slower than that of the wild type. However, metacaspase-silencing mutants Y58 and Y665 did not exhibit a more pronounced growth delay compared to the URA3-silencing mutants U534 and U594. These results indicate that the observed slow-growth phenotype is likely attributable to URA3 silencing rather than metacaspase deficiency (Figure S10).

### 3.8. Glmca1 Silencing Delayed Fungal Apoptosis and Enhanced Cell Viability in G. lucidum

**Figure 6:**
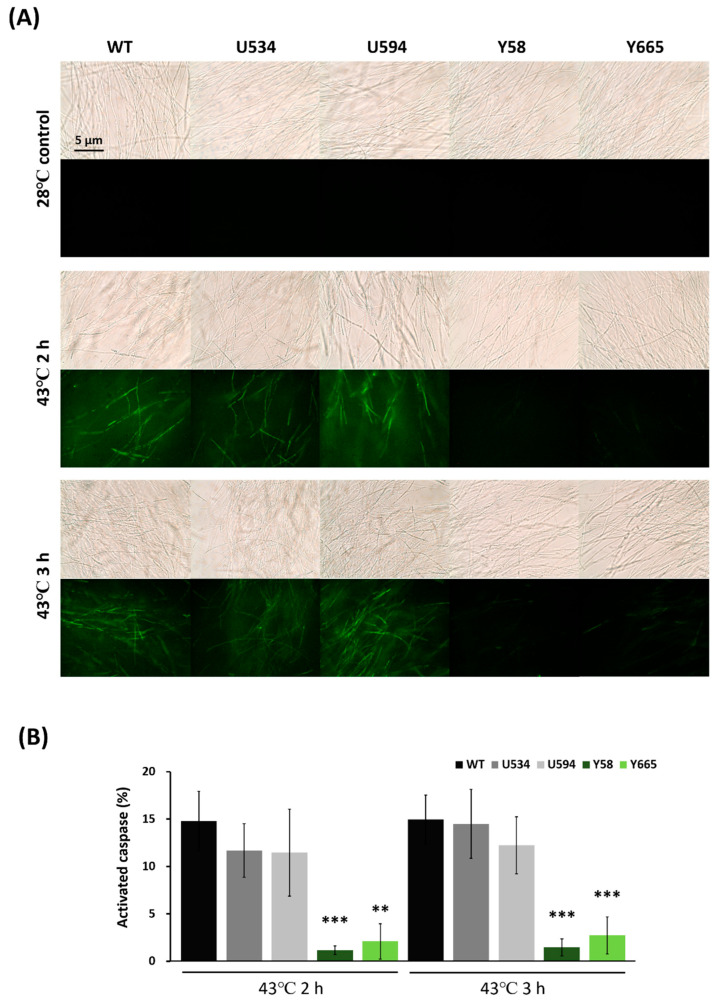
Caspase activity in *Ganoderma lucidum Glmca1*-silencing mutants. Mycelia were subjected to heat stress at 43 °C for 2 or 3 h. (**A**) Representative images of activated caspase-like activity in the wild type (WT) and mutant strains following heat treatment, detected using the CaspACE™ FITC-VAD-FMK in situ marker. (**B**) Quantification of mycelia exhibiting green fluorescence from eight independent images. Data are presented as activated caspase activity (%) relative to the 28 °C control. Values represent mean ± SD. Statistical significance is indicated as ** *p* < 0.01 and *** *p* < 0.001 compared with the control treatment. *Glmca1*-silencing mutants (Y58 and Y665) showed significantly lower caspase-like activity compared to the wild type and URA3-silencing controls (U534 and U594).

Caspase activity was evaluated in mutants Y58 and Y665. Following 43 °C treatment for 2 or 3 h, the area of green fluorescence representing active caspases was quantified. Caspase activity in Y58 and Y665 was significantly reduced compared to the wild type and URA3 controls (Figure 6A,B).

**Figure 7:**
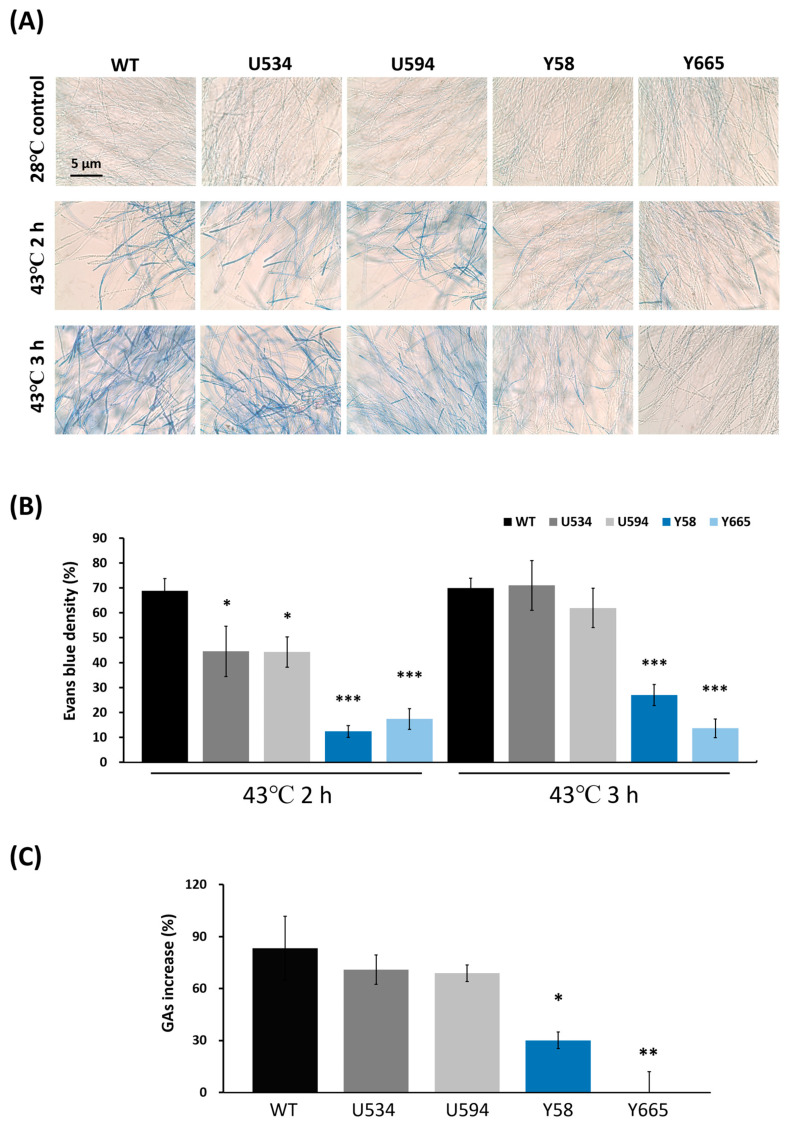
Cell viability and ganoderic acids (GAs) production in *Ganoderma lucidum Glmca1*-silencing mutants. Mycelia were subjected to heat stress at 43 °C for 2 or 3 h. (**A**) Cell viability in the wild type (WT) and gene-silencing mutants was analyzed using Evans blue staining. (**B**) Quantification and statistical analysis of Evans blue-stained mycelia from six independent images. *Glmca1*-silencing mutants (Y58 and Y665) exhibited significantly higher cell viability compared with the wild type and gene-silencing controls (U534 and U594). Data are presented as Evans blue density (%) relative to the 28 °C control. (**C**) GAs production in the wild type and gene-silencing mutants after 43 °C treatment for 6 h. Values represent the mean ± SD (n = 3). Data are presented as GAs increase (%) relative to the 28 °C control treatment. Statistical significance is indicated as * *p* < 0.05, ** *p* < 0.01, *** *p* < 0.001 compared with the control group.

Evans blue staining was used to assess cell viability in metacaspase-silencing mutants following apoptosis induction. The percentage of blue-stained mycelia relative to total mycelia was calculated after 43 °C treatment for 2 or 3 h. Compared to the wild type and URA3 control mutants U534 and U594 (with 60% and 68% Evans blue-stained mycelia after 2 and 3 h, respectively), metacaspase-silencing mutants Y58 and Y665 exhibited significantly lower staining values (12.4% and 27.3%, 17.4% and 13.7% after 2 and 3 h, respectively) (Figure 7A,B). Together, these results indicate that downregulation of metacaspase enhances cell viability and delays apoptosis in *G. lucidum*.

### 3.9. Glmca1 Silencing Reduced GAs Production During Fungal Apoptosis in G. lucidum

Reducing metacaspase expression not only enhanced cell viability but also delayed apoptosis in the mutants. We next investigated whether this delay affects GAs production. Under 43 °C treatment, GAs biosynthesis in mutants Y58 and Y665 (with increase rates of 30.1% and 0.2% after 6 h, respectively) was significantly lower than in the wild type and control mutants U534 and U594 (increase rates of 83.2%, 70.9%, and 68.8%, respectively) (Figure 7C). Mycelial weight decreased under heat stress in both wild type and mutant strains (Figure S11), with the biomass reduction in *Glmca1*-silencing mutants comparable to that in *Bcl-2* overexpression mutants. These results indicate that downregulation of metacaspase not only delays apoptosis but also reduces GAs production, further supporting the conclusion that apoptosis regulates GAs biosynthesis in *G. lucidum*.

## 4. Discussion

*G. lucidum*, a basidiomycete fungus, is widely used in Asia as both a medicinal fungus and a functional food. GAs, the major bioactive components of *G. lucidum*, exhibit diverse health-promoting effects [43,44]. While apoptosis has been linked to secondary metabolite production in fungi and plants [29,32], direct evidence demonstrating its regulatory role remains lacking. In this study, we introduce a non-invasive physical approach, heat-induced apoptosis, to enhance GAs production, thereby avoiding the contaminants associated with chemical or biological inducers. To determine whether apoptosis modulates secondary metabolite production in *G. lucidum*, we employed both *Bcl-2* overexpression and metacaspase silencing to block apoptosis signaling. Our results demonstrate that both approaches significantly delayed apoptosis, which was consistently accompanied by a reduction in GAs production. These findings provide definitive evidence that apoptosis functions as a crucial regulatory mechanism for GAs production in *G. lucidum*, offering a promising biotechnological strategy for the efficient production of bioactive fungal metabolite.

Environmental stress, nutrient limitation, and chemical or biological elicitors are primary inducers of secondary metabolite production in fungi and plants [45,46]. Among these, heat stimulation is commonly employed to study metabolite biosynthesis. For instance, heat shock (42–50 °C) induces trehalose production in *S. cerevisiae*, *Candida albicans*, and *A. nidulans* [47,48,49]. In this study, we show that GAs production in *G. lucidum* can be triggered by short-term heat treatments at 43 °C or long-term at 40 °C. Longer treatments at 42 °C for 6–12 h also increase GAs production [50]. Similarly, in other fungi, mild heat stress induces secondary metabolite biosynthesis over longer periods; for example, aflatoxin production in *A. flavus* occurs at 29–32 °C for 24 h [51]. These observations suggest that higher temperatures can induce metabolite production more rapidly but carry a higher risk of fungal lethality, whereas mild heat is safer but requires prolonged exposure to activate biosynthetic pathways. Consistent with our previous findings using chemical inducers, such as aspirin and cyclic AMP [31,32], both chemical- and heat-induced apoptosis are positively correlated with enhanced GAs production. Given that GAs are the primary bioactive constituents of *G. lucidum* for human health applications, the presence of chemical residues—often requiring rigorous downstream removal—poses a significant challenge for the development of functional food products. Consequently, physical heat treatment offers a more practical, sustainable, and contamination-free alternative for large-scale commercial production compared to traditional chemical induction methods.

Phosphatidylserine (PS) externalization and caspase-like activity are key features of apoptosis [52,53]. In this study, we measured PS externalization and caspase activity to evaluate apoptosis induced by heat treatment in *G. lucidum*. Under 43 °C treatment, caspase activity was detectable at 2 h and increased further by 3 h, with similarly high activity observed at 45 °C. PS externalization was detectable after 2 h at 43 °C and was initially accompanied by weak PI staining. Both PS externalization and PI staining increased with prolonged treatment. Although stimulation at 45 °C further increased the population of cells with externalized PS, PI staining also became stronger, while caspase activity at 3 h post-treatment did not significantly increase compared to 43 °C (Figure S1). PI, a membrane-impermeant DNA intercalating dye, distinguishes late-apoptotic/necrotic cells from early apoptotic cells. PI only stains cells with compromised membranes, such as necrotic or late apoptotic, whereas healthy or early apoptotic cells remain unstained. Our results suggest that apoptosis in *G. lucidum* begins at 2 h and increases by 3 h at 43 °C, whereas late apoptosis appears at 3 h under 45 °C treatment. Similar heat-induced apoptosis conditions have been reported in other fungi, including *A. nidulans* at 42 °C [54], *R. oryzae* at 42 °C [27], and *C. trifolii* at 55 °C [55].

To further investigate whether activation of apoptosis signaling regulates GAs production in *G. lucidum*, we examined the roles of key apoptosis regulators—mammalian Bcl-2 and *G. lucidum* metacaspase—through heterologous overexpression and gene silencing, respectively. In mammals, Bcl-2 family proteins are central regulators of the intrinsic apoptotic pathway [7,56]. Overexpression of human *Bcl-2* in *G. lucidum* enhanced cell viability, delayed apoptosis, and suppressed GAs production under heat stress. Similar effects of human *Bcl-2* overexpression have been reported in the ascomycetes *S. cerevisiae* and *C. gloeosporioides*, where it increased cell survival and delayed apoptosis [16,57,58]. These findings suggest that human Bcl-2 functions as a conserved regulator of fungal apoptosis across both Ascomycetes and Basidiomycetes.

Caspases are the primary cysteine proteases responsible for executing apoptosis in animals. Metacaspases are the functional orthologs of mammalian caspases in plants, fungi, and protists, although they exhibit distinct substrate specificities compared to caspases [9,11]. Metacaspases belong to the C14 protease family, which is composed of a p20 subunit and a p10 subunit, with the p20 subunit containing the catalytic His-Cys dyad. Based on domain architecture, metacaspases are classified into Types I, II, and III [20]. Fungal metacaspases are predominantly Type I, featuring a C-terminal metacaspase domain, sometimes accompanied by an N-terminal zinc finger motif and a proline-rich prodomain [20,21,59].

In *G. lucidum*, we identified a metacaspase gene, *Glmca1*, which belongs to the C14 protease family based on conserved domain analysis. Glmca1 exhibits the characteristic features of a Type I metacaspase, including a long N-terminal proline-rich prodomain followed by a P20 domain containing the catalytic His–Cys dyad. Furthermore, multiple sequence alignment placed Glmca1 within the Type I metacaspase clade, showing close homology with metacaspases from other Agaricomycetes fungi. Notably, a study by Jeong et al. demonstrated that substitution of the cysteine residue in the His-Cys catalytic dyad of the p20 subunit in *C. albicans* markedly reduced intracellular caspase activity, highlighting the essential role of this cysteine in metacaspase function [60].

Fungal metacaspases play a critical role in responding to environmental stress [21]. Disruption of metacaspase genes inhibits apoptosis in *S. cerevisiae*, *C. albicans*, and *Ustilago maydis* SG200 [24,61,62] and leads to growth defects in *A. fumigatus* and *Podospora anserina* [22,33]. Using a gene-silencing approach to downregulate *Glmca1* expression in *G. lucidum*, we found that the mutants showed no significant difference in growth compared to the wild type. However, the *Glmca1*-silencing mutants exhibited significantly higher cell viability and reduced caspase activity. These results indicate that *Glmca1* metacaspase is a key regulator of apoptosis in *G. lucidum*.

The role of metacaspases in fungal secondary metabolite production has been little explored, with the exception of trehalose synthesis in *C. albicans*. In *C. albicans*, trehalose, a non-reducing disaccharide, plays a critical role in protecting cells against oxidative stress [63]. Disruption of trehalose biosynthesis increases apoptosis under H_2_O_2_ treatment [64]. Conversely, *C. albicans* mutants lacking metacaspase exhibit reduced apoptosis and decreased metacaspase activity under H_2_O_2_ stress, while simultaneously showing elevated trehalose production [65], suggesting that trehalose functions as a protective metabolite against apoptosis.

GA biosynthesis in *G. lucidum* is a complex secondary metabolic process that follows the mevalonate (MVA) pathway to produce lanostane-type triterpenoids. Squalene synthase and lanosterol synthase are involved in the formation of squalene and lanosterol, respectively. Diverse GAs are synthesized from lanosterol by cytochrome P450s (CYP450) [66,67]. Within the *G. lucidum* genome, a total of 219 CYP gene sequences and more than 600 regulatory proteins have been identified [66]. Transcription factors such as CRZ1, GlSwi6, GCN4, Skn7, PacC, and MADS1 have been demonstrated to control GA production [67,68]. The functional characterization of CYP450s and regulators of GA biosynthesis is currently under investigation [68,69,70,71]. In our previous studies regarding aspirin- and cAMP-induced apoptosis and GAs production in *G. lucidum*, the expression of squalene synthase and lanosterol synthase was found to be reduced during apoptosis [31,32]. Therefore, we propose that apoptosis may upregulate GA biosynthetic genes downstream of lanosterol biosynthesis. The molecular mechanisms by which apoptosis promotes ganoderic acid biosynthesis—specifically regarding the control of CYP genes and transcription factors—warrant further investigation.

Ganoderic acids (GAs) are a specific subgroup of lanostane-type triterpenoids and are unique secondary metabolites found almost exclusively in fungi of the genus Ganoderma, most notably *G. lucidum*. While most triterpenes are based on tetracyclic (four-ring) or pentacyclic (five-ring) structures, GAs are characterized by a highly oxygenated lanostane skeleton. Their classification as ‘acids’ stems from the presence of at least one carboxylic acid group (–COOH), typically located at the terminus of the side chain. They also frequently feature multiple hydroxyl (–OH) and carbonyl (=O) groups, which contribute to their diverse pharmacological profiles. Over 400 secondary metabolites have been isolated from different strains of *G. lucidum*, more than 150 of which are lanostane-type triterpenoids [72]. Our extraction and HPLC assay system strictly followed the methods developed by Shiao’s studies [41,73], which focused on the isolation and determination of oxygenate triterpenoids in different *G. lucidum* strains, including the BCRC 36111 used in this study. Using this system, Shiao’s group previously identified 25 oxygenated triterpenes from *G. lucidum* [41], all of which were classified as GAs. The HPLC profiles of these strains were further characterized, revealing that the majority of the peaks corresponded to GAs [41]. Therefore, we assume that most of the HPLC peaks detected using this system correspond to GAs. For convenience, we use the term ‘total GAs’ to refer to the metabolites detected by this extraction–HPLC assay system. Additionally, we previously identified one of these peaks as ganoderic acid 24 (GA24); lanosta-7,9 (11), 24-trien-3α-o1-26-oic acid [42], which was reported in Shiao’s studies [41,73]. This confirms that we successfully reproduced the extraction–HPLC assay system and effectively detected the GAs produced by *G. lucidum* BCRC 36111. However, we cannot definitively conclude that all HPLC peaks are ganoderic acids. The presence of non-ganoderic compounds, such as non-lanostane-type triterpenoids or other metabolites, cannot be excluded. These compounds may also be regulated during heat-induced apoptosis and remain to be identified in future studies.

## 5. Conclusions

This study introduces a novel, non-invasive strategy for enhancing ganoderic acids (GAs) production in *Ganoderma lucidum* via heat-induced apoptosis. This physical induction method bypasses the contamination risks associated with chemical or biological additives in functional food production. To elucidate the regulatory role of apoptosis, we overexpressed the human anti-apoptotic gene Bcl-2 and silenced the endogenous Type I metacaspase Glmca1 in *G. lucidum*. Both genetic modifications successfully increased mycelial cell viability and delayed apoptosis under heat stress. Notably, this suppression of apoptotic signaling resulted in a significant reduction in GAs yield. Our findings provide compelling evidence that apoptosis functions as a critical regulatory switch for secondary metabolite production. This research establishes a mechanistic link between programmed cell death and GAs production, offering a ‘clean’ biotechnological approach for the manufacture of bioactive fungal metabolite.

## Data Availability

The original contributions presented in this study are included in the article/Supplementary Material. Further inquiries can be directed to the corresponding authors.

## Supplementary Materials

The following supporting information can be downloaded at: https://www.mdpi.com/article/10.3390/jof12050364/s1. Table S1: Metacaspases used in the phylogenetic analysis. Supplementary Materials S1: URA3 cDNA sequence of *Ganoderma lucidum* BCRC36111. Supplementary Materials S2: *Glmca1* cDNA sequence of *Ganoderma lucidum* BCRC36111. The underlined sequence was used for gene silencing. Table S2: Primers used in this study. Figure S1: Caspase activity and phosphatidylserine (PS) externalization assays in *Ganoderma lucidum* after heat treatment at 43 °C for 2 or 3 h, or at 45 °C for 3 h. Figure S2. GAs production and fungal growth of *Ganoderma lucidum* after heat treatment at 28, 35, 40 and 45 °C for 2 days. Figure S3: HPLC profiles and the fold changes in the major peaks for total ganoderic acids (GAs) of *Ganoderma lucidum* BCRC36111 under treatment at heat stress. Figure S4: Construction of the *Bcl-2* expression cassette in *Ganoderma lucidum* and PCR screening of transformants. Figure S5: Growth and colony morphology of *Bcl-2* overexpression strains. Mycelial agar plugs were cultured on PDA for 5 days. Figure S6: Biomass of the wild type (WT) and *Bcl-2* overexpression mutants (II-7, II48-3, and II104-2) after heat stress at 43 °C for 4 or 6 h. Figure S7: Gene structure, protein domains, and metacaspase signatures of *Glmca1* in *Ganoderma lucidum* BCRC36111. Figure S8: Constructs for *Glmca1* silencing in *Ganoderma lucidum* and PCR screening of transformants. Figure S9: Semi-quantitative RT-PCR analysis of gene expression in mutant lines. Figure S10: Growth and colony morphology of *Glmca1*- (Y58 and Y665) and *URA3*- (U534 and U594) silencing strains. Figure S11: Biomass of the wild type (WT), *URA3*-silencing strains (U534 and U594), and *Glmca1*-silencing strains (Y58 and Y665) after heat stress at 43 °C for 6 h.
