# Supplementary material for: Heat-Stress Induced Apoptosis: A New Biotechnological Strategy to Enhance Ganoderic Acids Production in Ganoderma lucidum
Source: J Fungi (Basel). 2026 May 15;12(5):364. doi: 10.3390/jof12050364 (PMC13208671; doi:10.3390/jof12050364)
Supplement: Supplementary file 1 [file jof-12-00364-s001.zip › jof-4242087-supplementary.pdf]

Table S1. Metacaspases used in the phylogenetic analysis.

Type	Species	Protein name	Protein ID	Refference
Type I metacaspase	*Arabidopsis thaliana*	metacaspase 1	NP_171719	
	Arabidopsis thaliana	metacaspase 2	NP_001031711	
	Vitis vinifera	metacaspase 1	AGJ94048	
	Vitis vinifera	metacaspase 2	AGJ94049	
	Zea mays	metacaspase 1	NP_001152418	
	Aspergillus fumigatus	metacaspase A	DQ518209	[36]
	Candida albicans	metacaspase 1	orf19.5995 (from *Candida* genome database)	http://www.candidagenome.org/
	Colletotrichum gloeosporioides	metacaspase 1A	XP_045264054	
	Colletotrichum gloeosporioides	metacaspase 1B	XP_045265311	
	Coprinopsis cinerea	metacaspase 1	XP_001836373	
	Ganoderma adspersum	metacaspase 1	KAM5530406.1	
	Laccaria bicolor	metacaspase 1	XP_001875835	
	Magnaporthe oryzae	metacaspase 1	XP_003712417	
	Magnaporthe oryzae	metacaspase 2	XP_003716368	
	Pleurotus ostreatus	metacaspase 1	PPO01696	[33]
	Podospora anserina	metacaspase 1	Q874X7	
	Podospora anserina	metacaspase 2	CDP22321	
	Rhizoctonia solani	metacaspase 1	OP889286	
	Saccharomyces cerevisiae	metacaspase 1	Q08601	
	Ustilago maydis	metacaspase 1	NC_026479	
Type II metacaspase	*Arabidopsis thaliana*	metacaspase 8	NP_173092	
	Nicotiana tabacum	metacaspase 1	EU869285	
	Oryza sativa	metacaspase 1	AAT07577	
	Solanum lycopersicum	metacaspase 1	NP_001266198	[34]
	Triticum aestivum	metacaspase 4	JN807891	[35]
	Vitis vinifera	metacaspase 5	AGJ94052	

Supplementary Materials S1. ***URA3*** **cDNA sequence of** ***Ganoderma lucidum*** **BCRC36111.** The underlined sequence was used for gene silencing.

ATGGCATCCCCAGTAAAGAAAACATACGCCCAGAGGGCCGCCAAGCATCCCAACCCGGCCGCGAAAGCTCTCCTCGAGACCATCGA
GCGCAAGCGCACAAATCTGTCCGTCAGTGTTGACGTGACCAAACAAGAGGACTTCTTCAGAATCGTGGACACGGTCGGTCCATACGT
CTGTCTTGTAAAGACGTAGACATCATCGAGGATTTTAAGCCATCCGTCATCGAACGCCTCAAAACACTCAGCCAGAAGCATGACTTCC
TCATCAGGAATAGGAAACACGGTGGCGCTACAGTATTCTGCGGGCGTGCACAAGATCGCGAGCTGGTCGCACATCACCAACGCACA
CCCTGTTCCAGGACCTTCCATAATTTCCGGCCTCAAGTCTGTCGGTCTGCCCCTCGGTAGAGGGCTCTTGCTTCTCGCTGAGATGAGC
ACCAAGGGCAGCCTCGCGACTGGTCTGTACACGGAGGAGGCGGTCCGTATGGCTCGCGCCAACCGCGACTTCGTGATTGGCTTTATT
GCCCAGCAACGCATGGACGGCGTCGGTTTGCGGGAATGCGAGTCCTCGCCGGACGAAGACTTCCTGATCCTCACTCCAGGCGTCGG
TGACGCCAAAGGAGACAGCATGGGACAGCAATACCGAACGCCCAGAGAGGTGATTGTAGAATCCGGCTGCGATGTCATCATCGTTG
GCCGGGGCGTTTACGGAAAGGACAATGGCGCAAATACGGAGGAAGTCCGCACACAGGCGGAGAGGTACCGTACGGAAGGTGGAA
GGCATACCAGGAGAGAATCGGTATCTCTGACTAGTGCTATCATTGGGCAGAGCGGGTATAGCAGTACAGATCGCAGTAAACAAAAT
ATCCTACCGCTACAGACGTAG

Supplementary Materials S2. ***Glmca1*** **cDNA sequence of** ***Ganoderma lucidum*** **BCRC36111.** The underlined sequence was used for gene silencing.

ATGTGGAACAACTATCCTGGAGACCAAGGCGGTTATCGATCGGGCCCTCCTGGCGCGCCTGCTGGCTTTGGCTTTCCCACCCCGAACATGCCATCG
CCGACTCCTGGCTACAACCCCGGATACGCACCATCTTATGGGGCACCGCCTAGCGGCTATCCTCCGCCATCGGTTCCTGGCTACCCTCCGCCACCCG
GTCCTGGCTACCCTCCGCCGCCCGGTCCTCCGCCGCCTGGTGGTCACCAGCACCACCAGCACCACCAACATCACCAAGAAAGCGGCTACGGACCTC
CGCCTGGGGCACCTCCTGGTTACGGTGGGGCGCCTCCAATTCCAGGTGGACGACCTGACGGAAGCCGCTACGCGCCTCCGCCCGGCCCTCCTCCA
GGAGCTGGCTACGGCGGCTATCAGCCACCGCCTGGACCGCCCCCTCCCCCGCCCAGCAGTCAGCAAAATTATGGTCCGACGTTCATGGGTCGAGA
TAACCAACAACACCAACTTTTCTACCAGTACTCGCAATGTAATGGGAAGAAGAAGGCGCTTTGTATTGGTATCAACTACTTCAGACAGTCCGCGGA
GCTCAAAGGGTGTATCAACGACGCCCGCAACGTCCAAAACTTCCTCAAGCAGCTATGGGGCTATCGGGACGAAGACATAGTCATGTTGACGGATG
ACGCCCAGAACCCTCGCCAGATCCCTACTAGGGAAAATATCATTCAAGCTATGCAATGGCTCGTACGCAACGCGCAGCCCAATGATTCGTTGTTCT
TCCACTACTCGGGACATGGTGGTCAAACGAAGGACCTCGATGGAGATGAGGCGGATGGTTTTGACGAGGTCATTTATCCCGTTGACTTCGAGAAG
GCCGGACATATCGTCGACGACATGATGCACGATATCATGGTCAAGCCGCTCCCTCCTGGTTGTCGCCTCACTGCAATCTTTGATTCTTGCCACTCAG
GCTCTGTGCTTGATCTCCCTTACGTATACTCAACCGAGGGGAAGATCAAGGAGCCTAACTTGGCAGCCGAAGCCGGTCAAGGTGTCCTTTCTGCTG
TGACATCATACGCTCGTGGTGATATGGGTGGGGTATTCTCGTCTGTGAGCGGCCTCCTCAAGACGGCGACTGGCGGCACTCAGCGTGCCGAAAGG
AAAACACGCGCAACCAAGACTTCTCCTGCCGATGTCATCTCATGGAGCGGCTGCAAGGATTCTCAGACAAGTGCCGATACCTTCGAGGCTGGACA
GTCGACTGGCGCGATGAGCTATGCCTTTATGACGTGTCTACGCCAGAACAAGCAGCAGAGCTACCAACAGTTGCTTCAAAATATTCGGAGTATTTT
GAGGCAGAAGTACAGCCAGAAGCCCCAGCTCTCGAGTTCTCATCCCATGGACGTGAACTTGTTATTCATTTGC**TGA**

Table S2. Primers used in this study.

Primer	Primer name	Sequence (5’→3’)	Usage
P1	*URA3*-F	ATGGTGGCCGTGGCCAAGCAAACAT	*URA3* amplification
	URA3-R	CTAATCCGAGATCCCAACCCTTTCC	
P2	DP-*Yca1*_nest-YcaI-F	TCACGTCCATGGGATGAGAAC	*Glmca1* cloning
	DP-*Yca1*_nest-R	CGCAGCAGTCAGCAAAATTATGGTCCGA	*Glmca1* cloning and expression
P3	DP-*Yca1*_6nt-PmlI-XmaI-*Yca1*-F	CTTAAACACGTGCCCGGGTGCTGCTTGTTCTGGCGTAG	*Glmca*1 PCR and nested PCR
	DP-*Yca1*_6nt-SpeI-SbfI-*Yca1*-R	GAAGTCACTAGTCCTGCAGGATGGTCCGACGTTCATGGGT	
P4	6nt_XmaI-*eGFP*_F	CACGTGCCCGGGCTTGTAGAGTTCGTCCATACCG	*eGFP* amplification
	6nt_SpeI-*eGFP*_R	CAGAGAACTAGTATGGTGTCCAAGGGCGA	
P5	Ggpd_*bcl2*_F175	GCCTTGCAATTGGTGTTCGT	Screening of *Bcl-2* overexpression transformants
	Ggpd_*bcl2*_R1323	TTCATGACGATCTCGCGGTT	
P6	*bcl2* F10699	TATTTTCTCCTCGCAACCTG	*Bcl-2* expression and transformant screening
	bcl2 R10933	GTAAAAGGGGTGAGGTGGAG	
P7	*Bcl2*_ter_F1271	CGTGCGCTTACAGGTCAAAG	Screening of *Bcl-2* overexpression transformants
	*Bcl2*_ter_R2442	GAGCGGATTCCTCAGTCTCG	
P8	*Hyg*_In_F1510	CAGGATCTCCCTGAGACCGA	Amplification of hygromycin resistant gene
	*Hyg*_In_R1833	TTGTCGGTGAGGACGTTGTT	
P9	GL.*GPD*_QPCRR_1061	CCGTTGAGGCTGGGGATGAC	RT-PCR for GPD
	GL.*GPD*_QPCRF_959	CATGAAGGACTGGCGTGGT	
P10	New_GPD p_F	ATCTGCGATAACATCGGTCG	Screening of *Glmca1* and *URA3* silencing transformants
	YcaI seq_R	CGCCTCACTGCAATCTTTGA	Screening of *Glmca1* silencing transformants
P11	*YcaI*-DP_seq_F	GATCTTCCCCTCGGTTGAGT	
	DP-*URA3*-R1100	AACGCCCAGAGAGGTGATTG	
P12	RT-PCR_*URA3*_F	CAATCACCTCTCTGGGCGTT	
	URA3-35S-R1261	ACCTCCTCGGATTCCATTGC	Screening of *Glmca1* and *URA3* silencing transformants
P13	RT-*URA3*_B30F	TTGACGTGACCAAACAAGAGG	*URA3* RT-PCR
P14	RT-PCR_*URA3*_R	TTTCCGGCCTCAAGTCTGTC	
P15	NewRT-*URA3*-1_R	CTATACCCGCTCTGCCCAA	
P16	DP-*Yca1*_nest-F	CAACTGTTGGTAGCTTGCTGCTTG	*Glmca1* RT-PCR

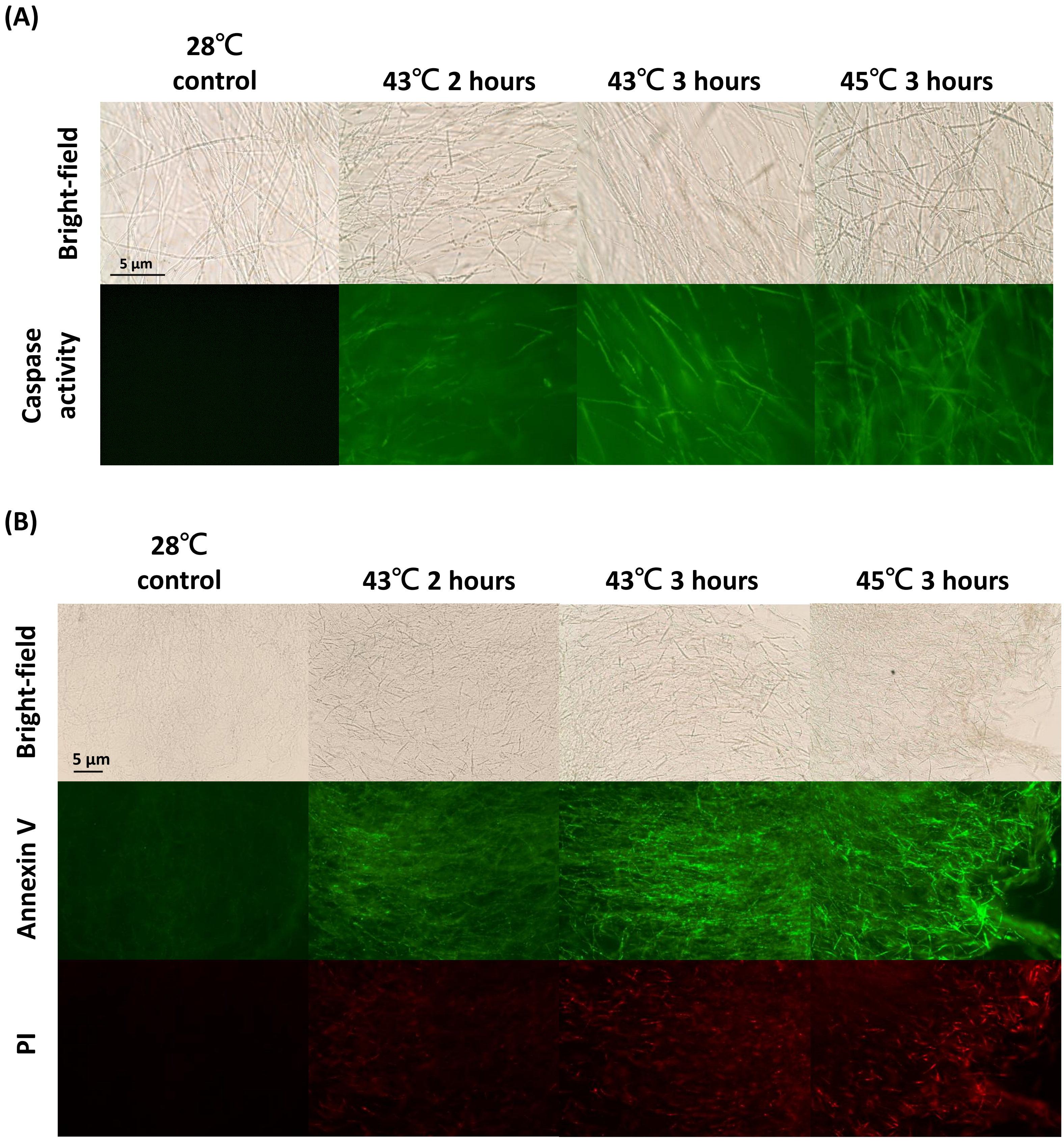

Figure S1. Caspase activity and phosphatidylserine (PS) externalization assays in *Ganoderma lucidum* after heat treatment at 43˚C for 2 or 3 h, or at 45˚C for 3 h. (A) Caspase activity was detected using the CaspACE™ FITC-VAD-FMK In Situ Marker. (B) PS externalization was analyzed by Annexin V staining with propidium iodide (PI) counterstaining. More than ten independent samples were analyzed, and representative images are shown.

(A)

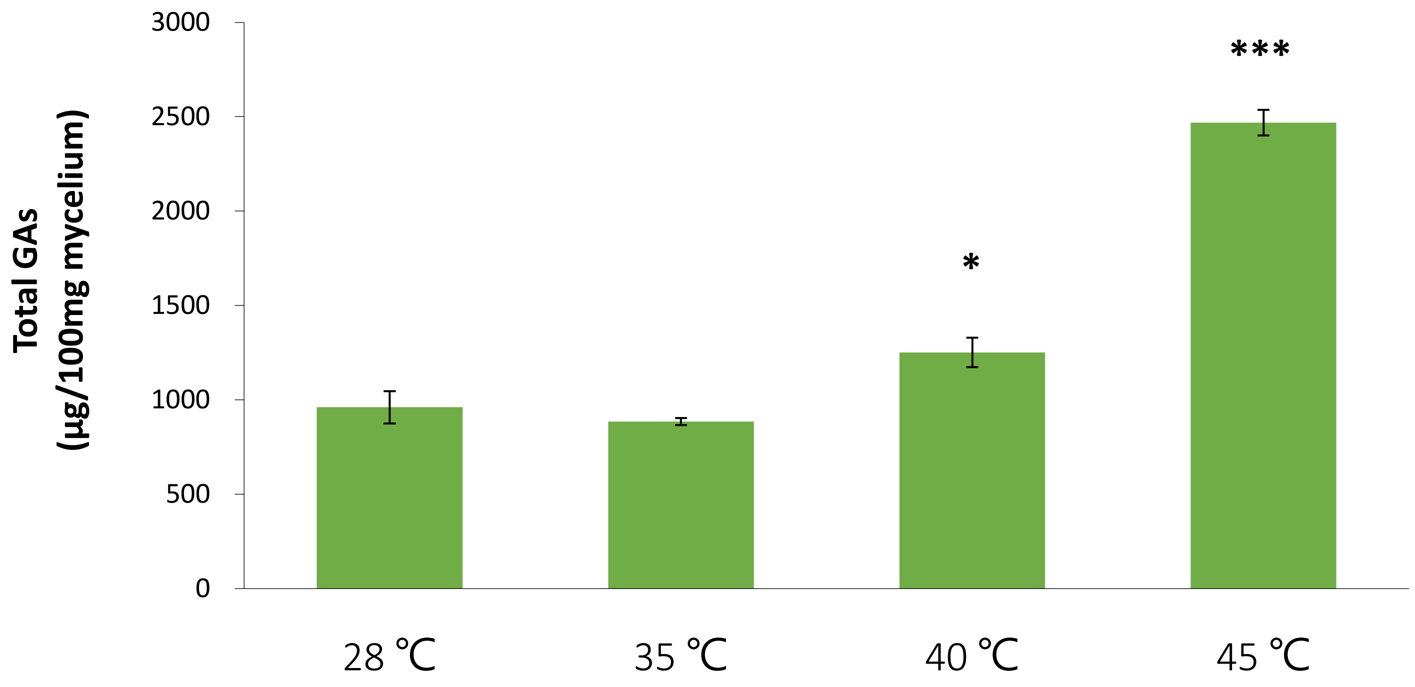

(B)

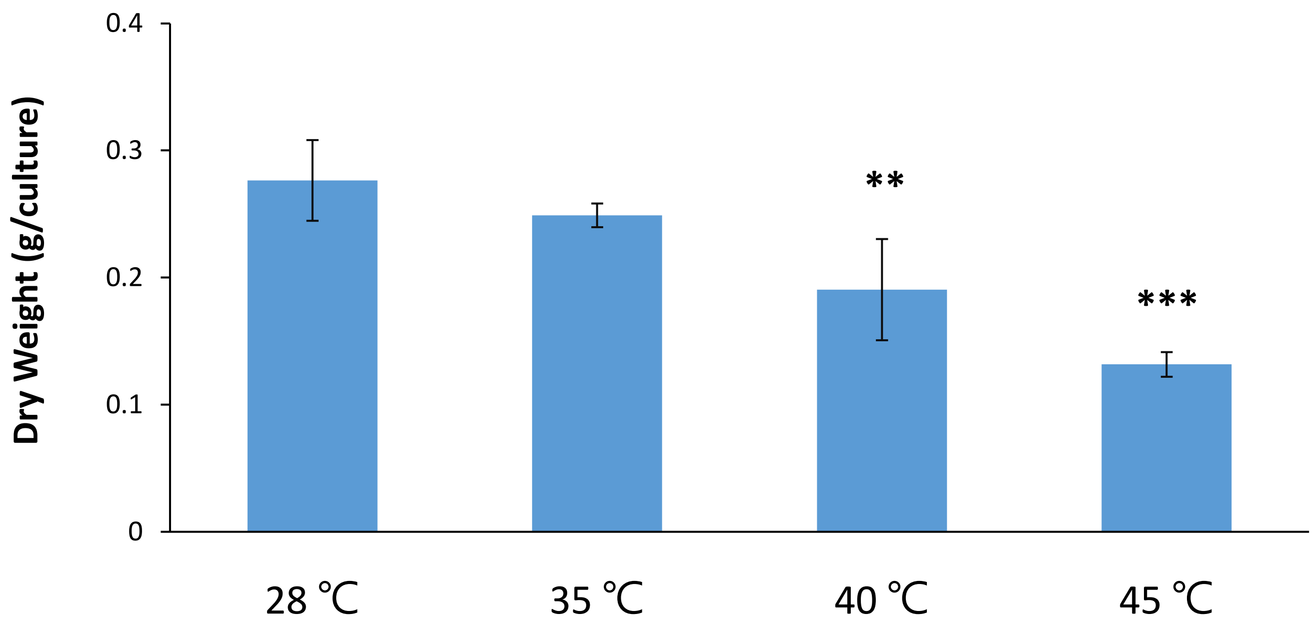

Figure S2. GAs production and fungal growth of *Ganoderma lucidum* after heat treatment at 28, 35, 40 and 45°C for 2 days. (A) GAs production (B) and mycelial dry weight were detected after treatments for two days. Three replicates were used to calculate mean and standard deviation. Values represent the mean ± SD (n = 3). Statistical significance is indicated as *$p < 0.05$, **$p < 0.01$, ***$p < 0.001$ compared with the control.

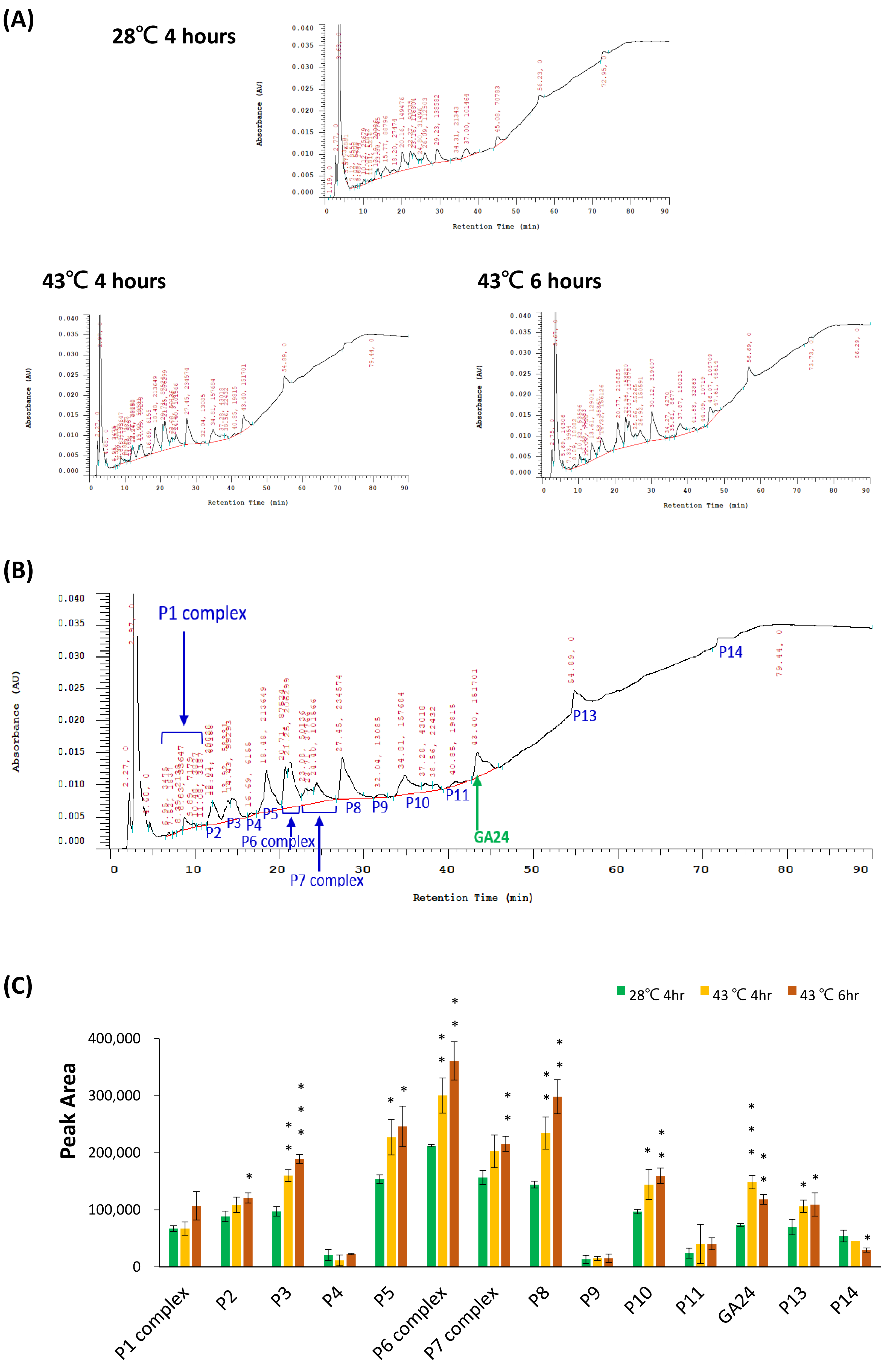

Figure S3. HPLC profiles and the fold changes of the major peaks for total ganoderic acids (GAs) of *Ganoderma lucidum* BCRC36111 under treatment at heat stress. (A) HPLC profiles of samples treated at 28 ˚C (control) for 4 h, or 43 ˚C for 4 h and 6 h. (B) The peak numbers are indicated using 43 ˚C for 4 hr as the representative profile. (C) Fold changes of the major peaks. Data from three replicates were used to calculate mean and standard deviation. Statistical significance is indicated as *$p < 0.05$, **$p < 0.01$, ***$p < 0.001$ compared with the control.

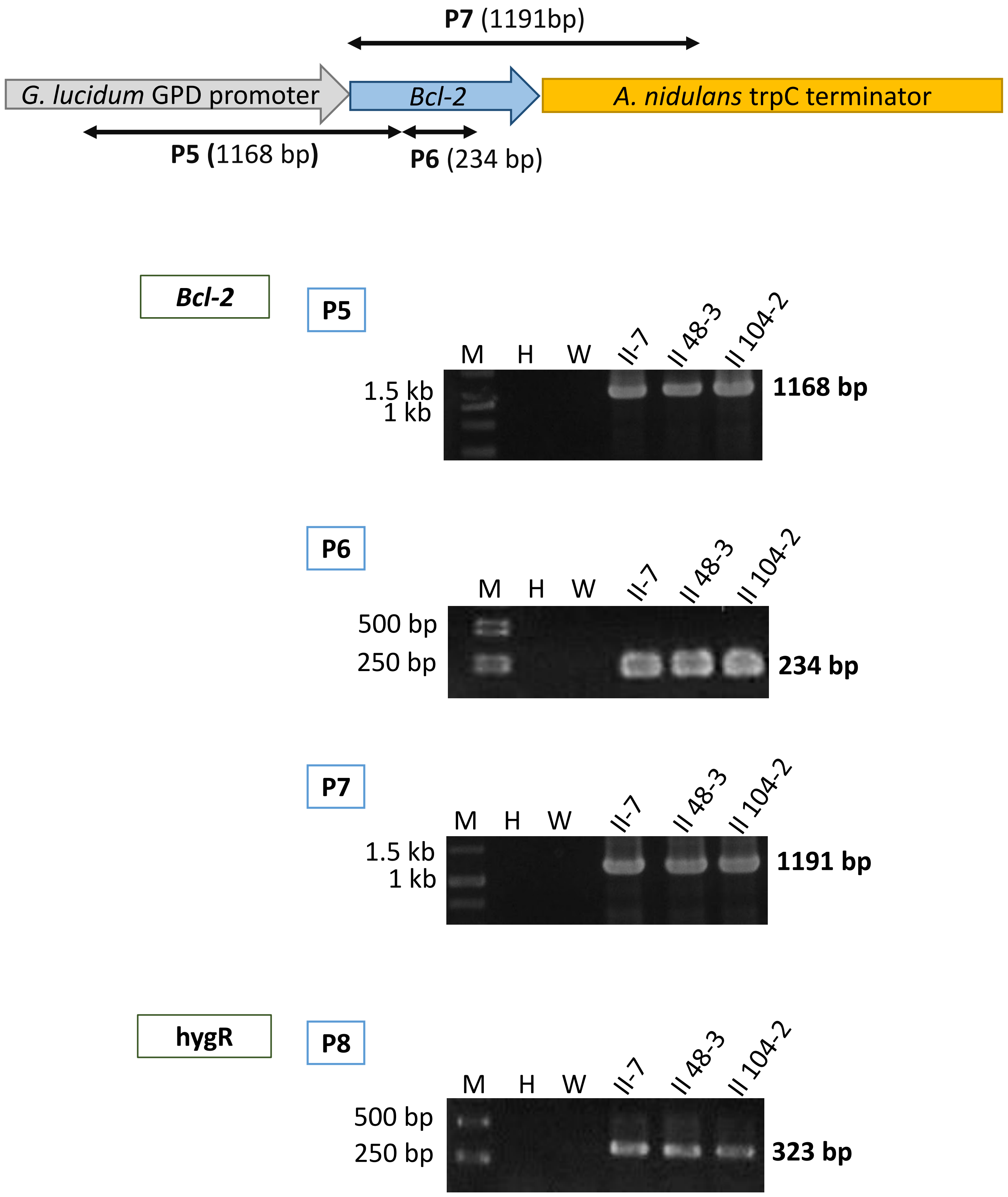

Figure S4. Construction of the *Bcl-2* expression cassette in *Ganoderma lucidum* and PCR screening of transformants. *Bcl-2* was driven by the *GPD* promoter, and transformants were verified by PCR using primers P5–P8 to amplify *Bcl-2* and *HygR*. M, marker; H, H_2O; W, wild-type

(A)

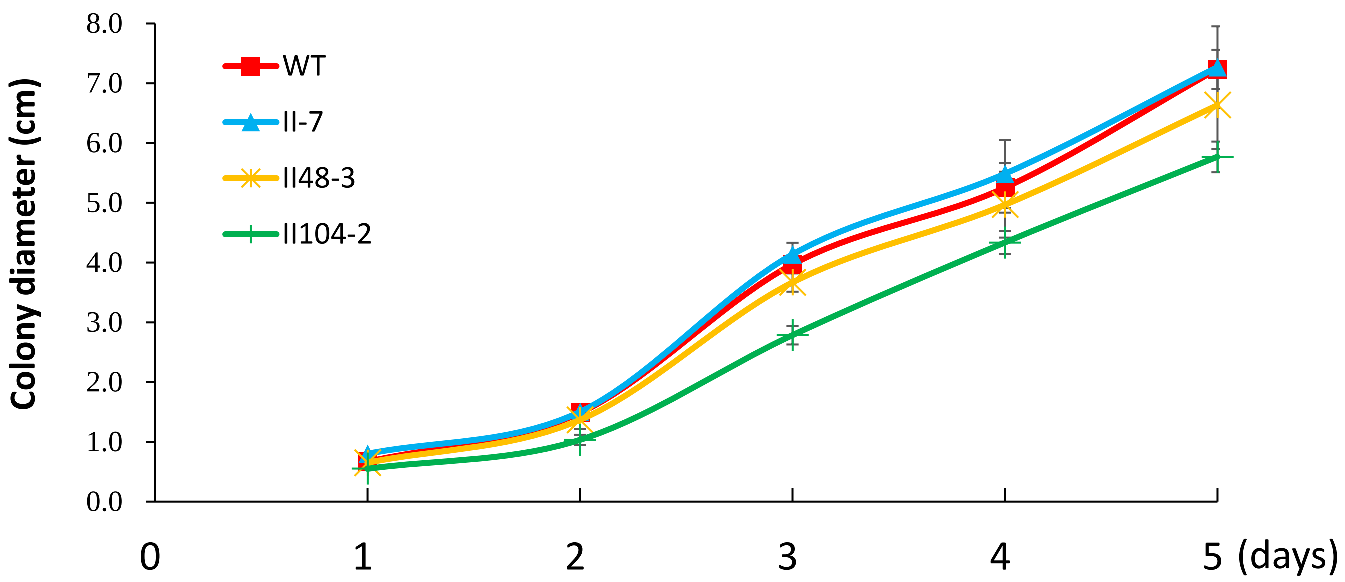

(B)

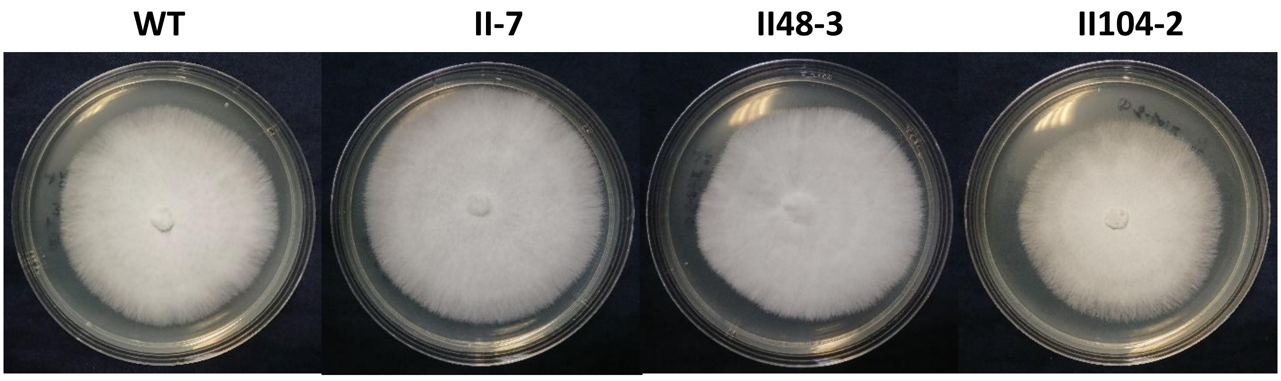

Figure S5. Growth and colony morphology of *Bcl-2* overexpression strains. Mycelial agar plugs were cultured on PDA for 5 days. (A) Colony diameters were measured and recorded daily. (B) Colony morphology was documented by photography on day 5. For each mutant, three biological replicates (n = 3) were used to calculate mean colony diameter and standard deviation. Notably, mutant line II104-2 exhibited phenotypic instability during subculturing, as reflected in the growth curve (A) and colony morphology (B). However, statistical analysis revealed no significant difference in growth between II104-2 and the wild-type (WT) strain.

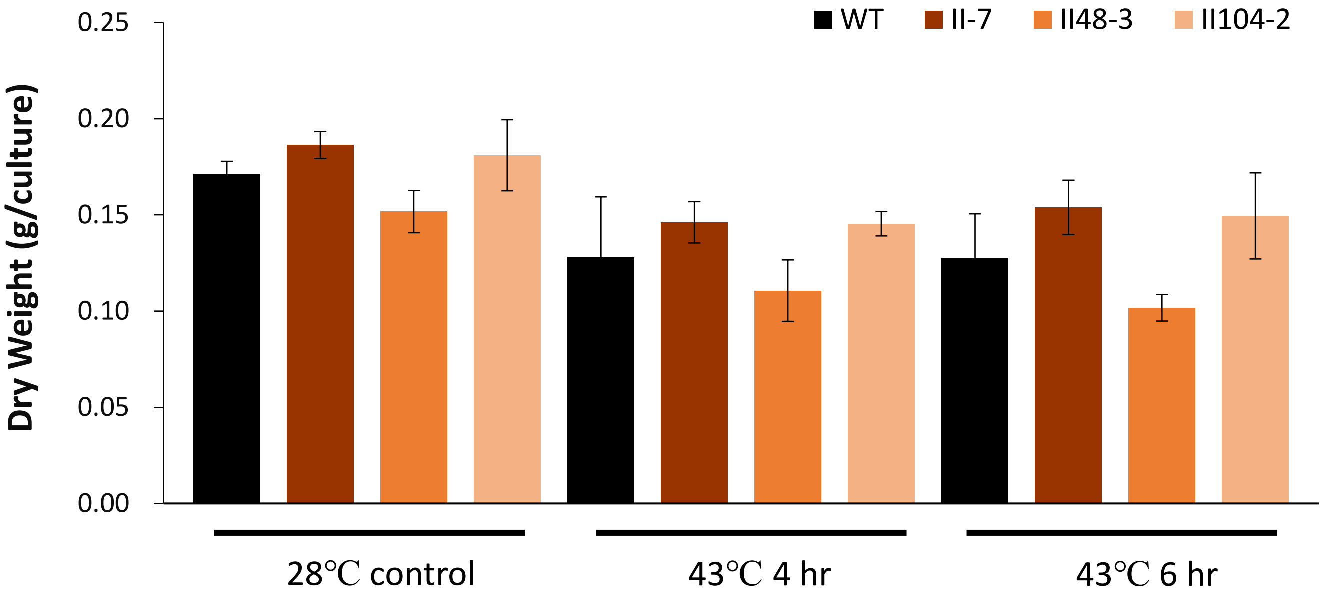

Figure S6. Biomass of the wild-type (WT) and *Bcl-2* overexpression strains (II-7, II48-3, and II104-2) after heat stress at 43 ˚C for 4 or 6 hours.

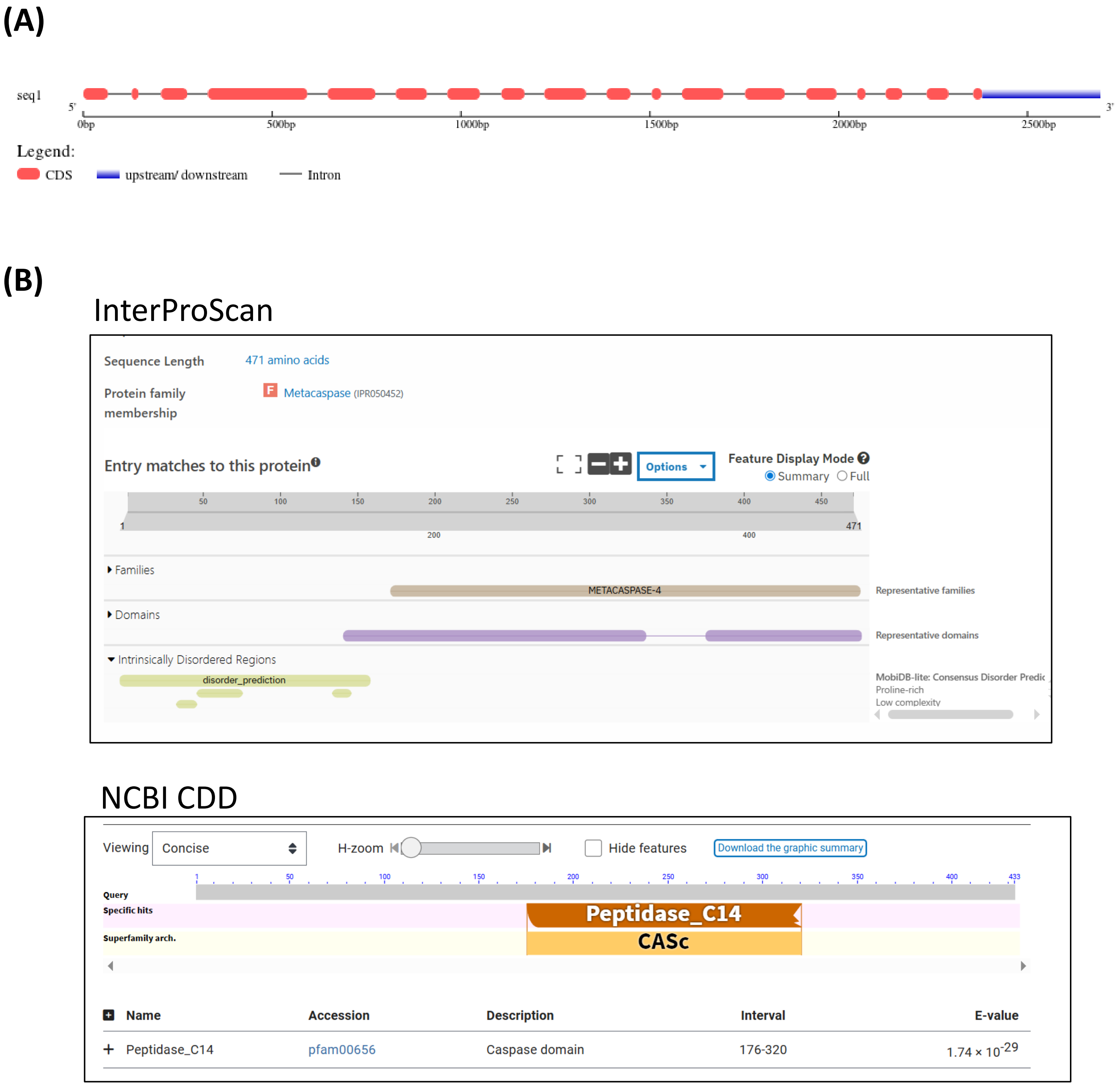
(A)
seq1
5'
3'
0bp
500bp
1000bp
1500bp
2000bp
2500bp
Legend:
CDS
upstream/ downstream
Intron
(B)
InterProScan
Sequence Length
471 amino acids
Protein family membership
Metacaspase (IPR050452)
Entry matches to this protein
Options
Feature Display Mode
Summary
Full
Families
METACASPASE-4
Representative families
Domains
Representative domains
Intrinsically Disordered Regions
disorder_prediction
MobiDB-lite: Consensus Disorder Predic
Proline-rich
Low complexity
NCBI CDD
Viewing
Concise
H-zoom
Hide features
Download the graphic summary
Query
Specific hits
Superfamily arch.
Peptidase_C14
CASc
Name
Accession
Description
Interval
E-value
Peptidase_C14
pfam00656
Caspase domain
176-320
1.74 × 10-29

(C)

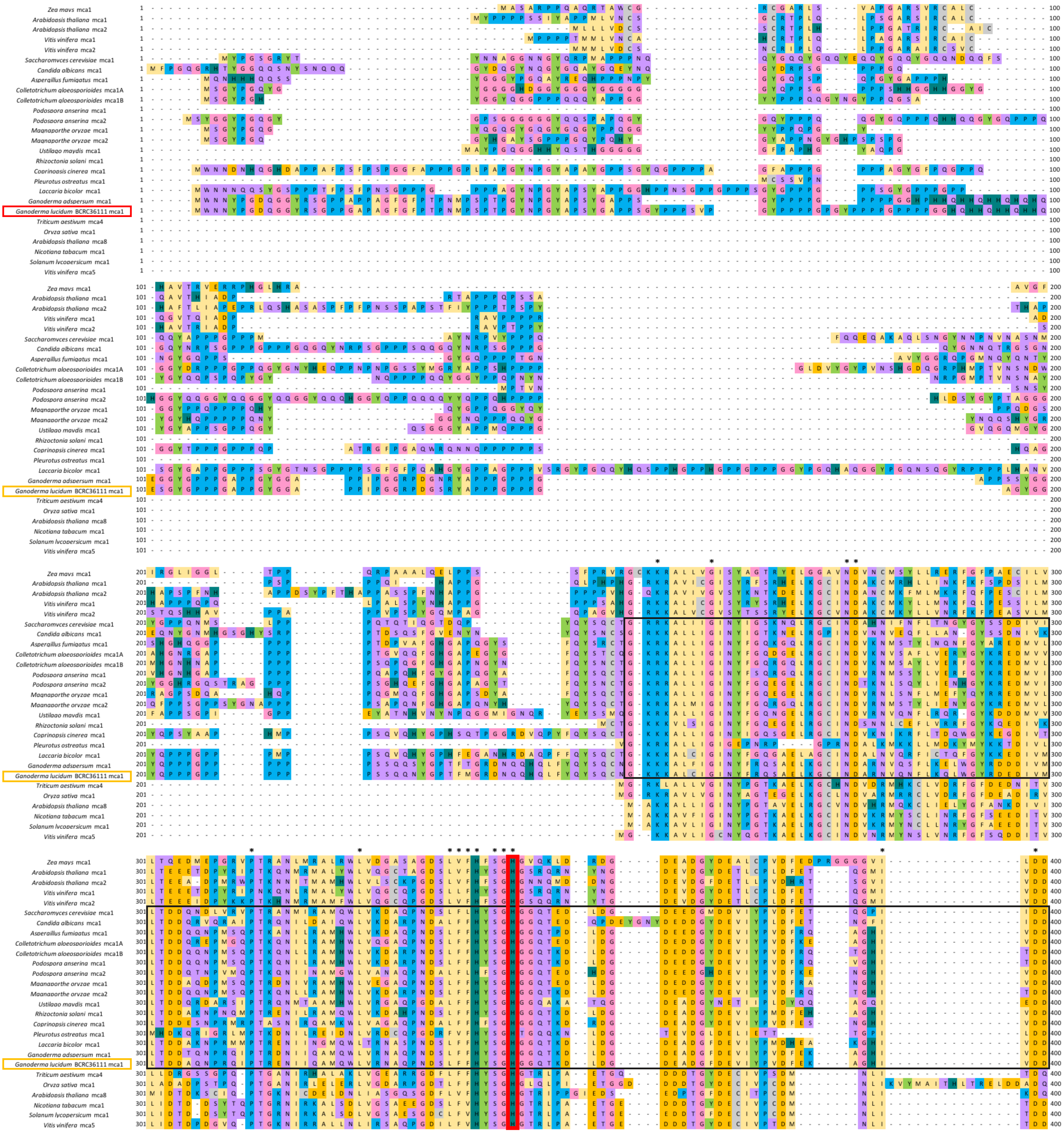
Zea mays mca1
Arabidopsis thaliana mca1
Arabidopsis thaliana mca2
Vitis vinifera mca1
Vitis vinifera mca2
Saccharomyces cerevisiae mca1
Candida albicans mca1
Aspergillus fumigatus mca1
Colletotrichum gloeosporioides mca1A
Colletotrichum gloeosporioides mca1B
Podospora anserina mca1
Podospora anserina mca2
Magnaporthe oryzae mca1
Magnaporthe oryzae mca2
Ustilago maydis mca1
Rhizoctonia solani mca1
Coprinopsis cinerea mca1
Pleurotus ostreatus mca1
Laccaria bicolor mca1
Ganoderma adspersum mca1
Ganoderma lucidum BCRC36111 mca1
Triticum aestivum mca4
Oryza sativa mca1
Arabidopsis thaliana mca8
Nicotiana tabacum mca1
Solanum lycopersicum mca1
Vitis vinifera mca5

(C) continued

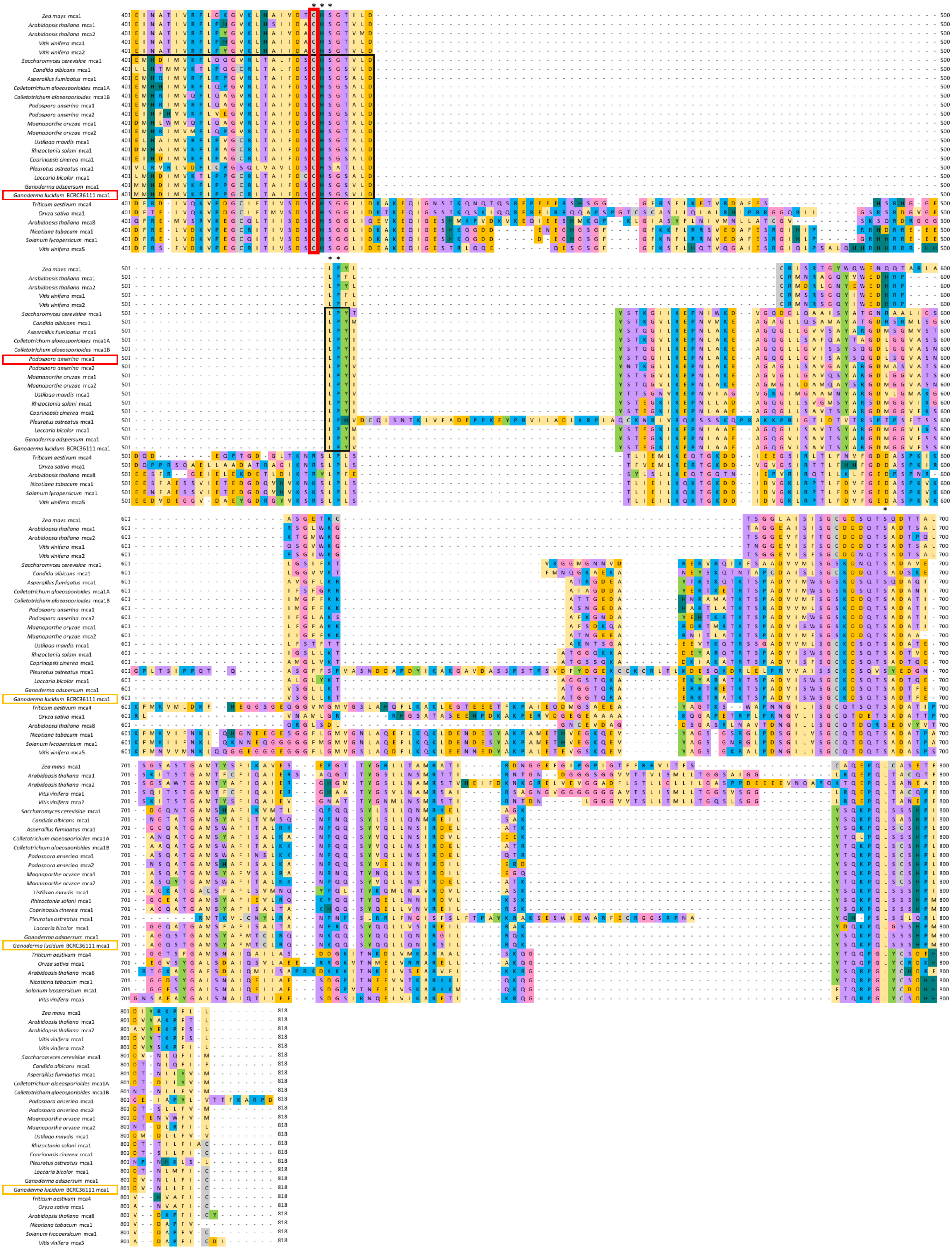

Figure S7. Gene structure, protein domains, and metacaspase signatures of *Glmca1* in *Ganoderma lucidum* BCRC36111. (A) Genomic organization of *Glmca1*, showing 18 exons (indicated in red).
(B) Protein domain analysis performed using InterProScan and the NCBI Conserved Domain Database (CDD), indicating that Glmca1 is a metacaspase belonging to the peptidase C14 family.
(C) Amino acid sequence alignment revealing the fungal p20 subunit (indicated by black boxes) and the conserved catalytic His–Cys dyad (highlighted by red boxes)- see next page.

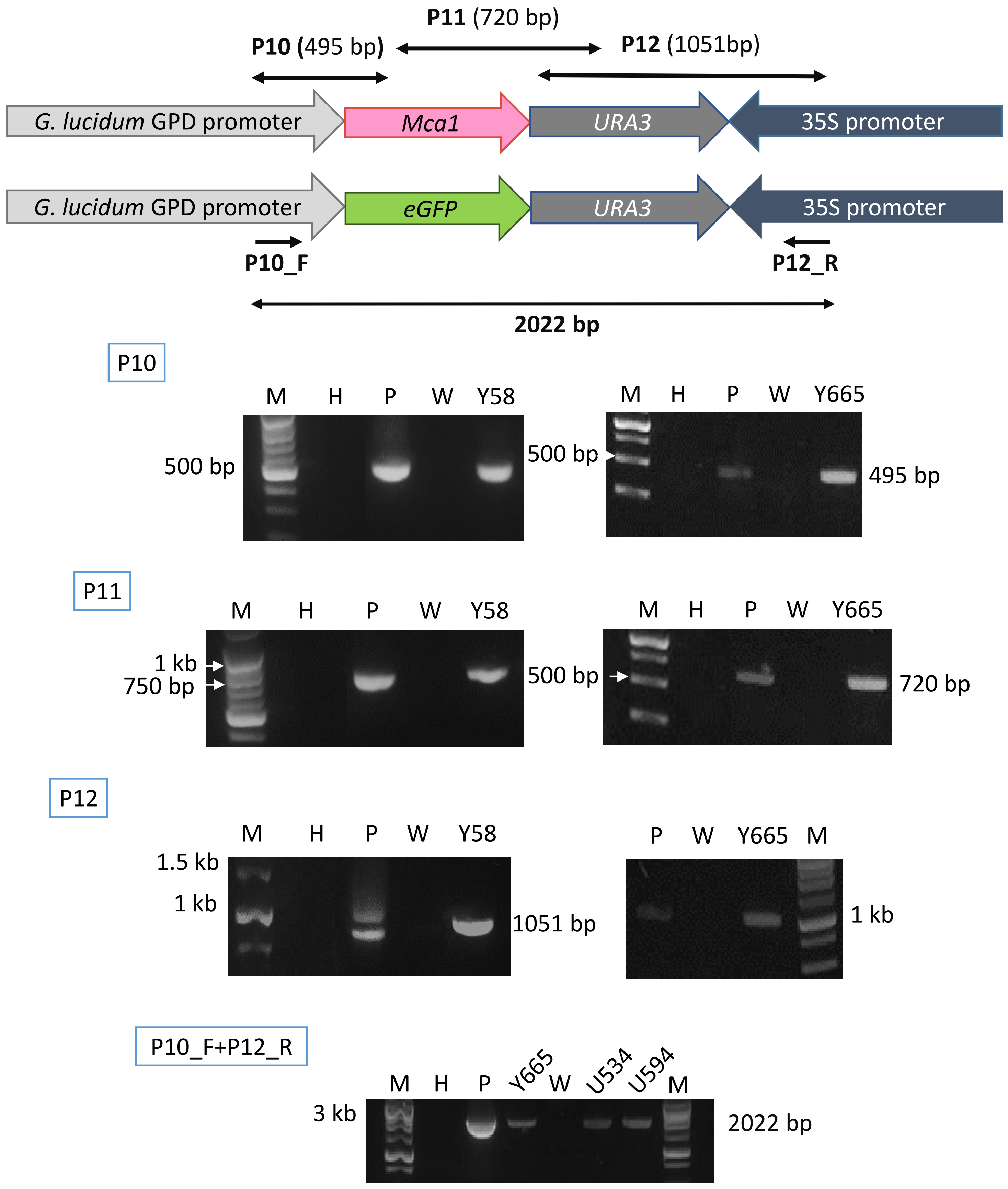

Figure S8. Constructs for *Glmca1* silencing in *Ganoderma lucidum* and PCR screening of transformants. Partial fragments of *Glmca1* and *URA3* or *eGFP* and *URA3* were placed under the control of the dual promoters GPD (from *G. lucidum*) and CaMV 35S, and the resulting constructs were transformed into *G. lucidum*. Transformants were screened by PCR analysis using the indicated primers. M, marker; H, H_2O; P, plasmid; W, wild-type.

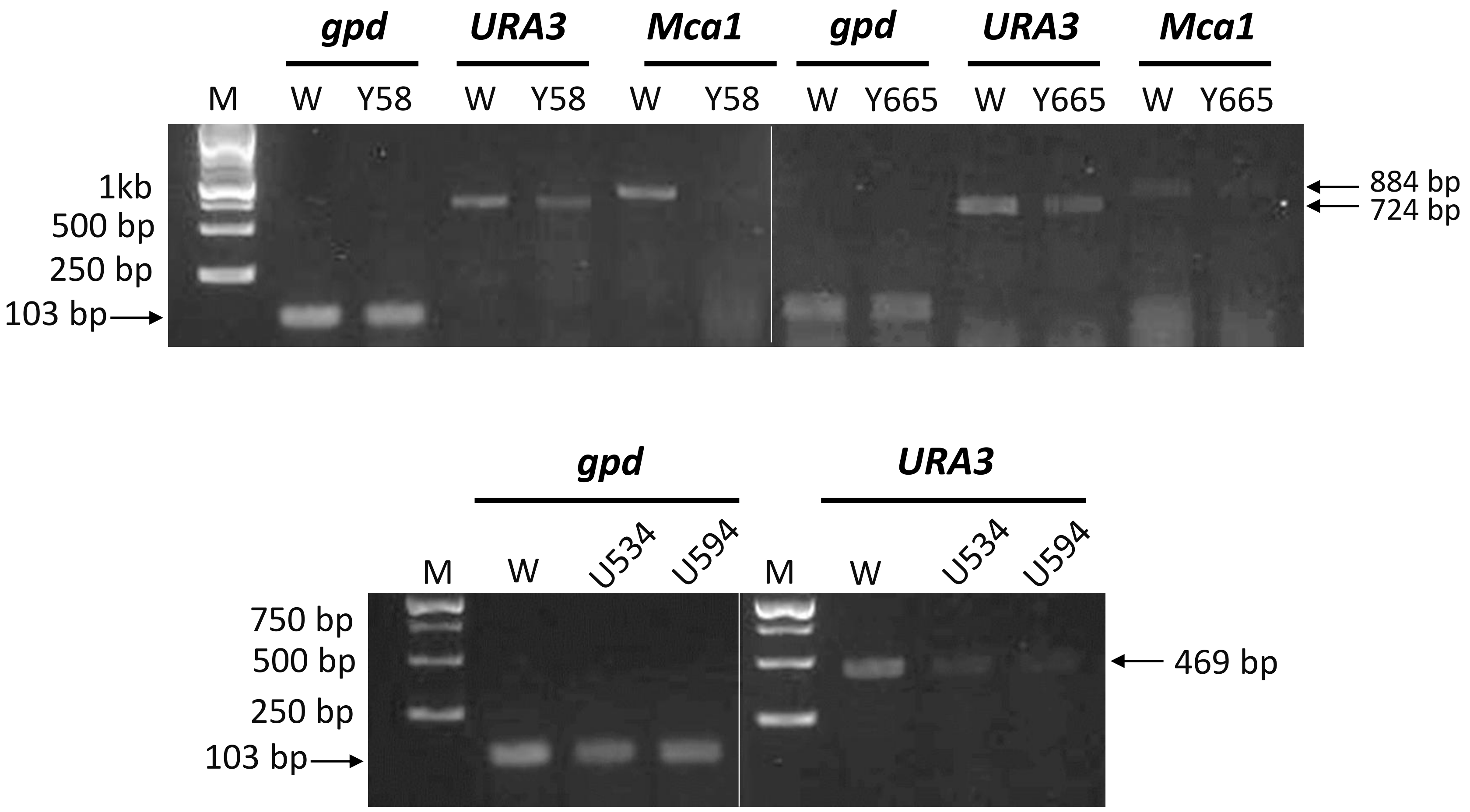

Figure S9. Semi-quantitative RT-PCR analysis of gene expression in mutant lines. (A) *URA3* transcript levels (469 or 724 bp) were analyzed in mutants U534, U594, Y58, and Y665. (B) *Glmca1* expression (884 bp) was examined in silencing mutants Y58 and Y665. The *gpd* gene (103 bp) was used as an internal control. M, DNA marker; W, wild type.

(A)

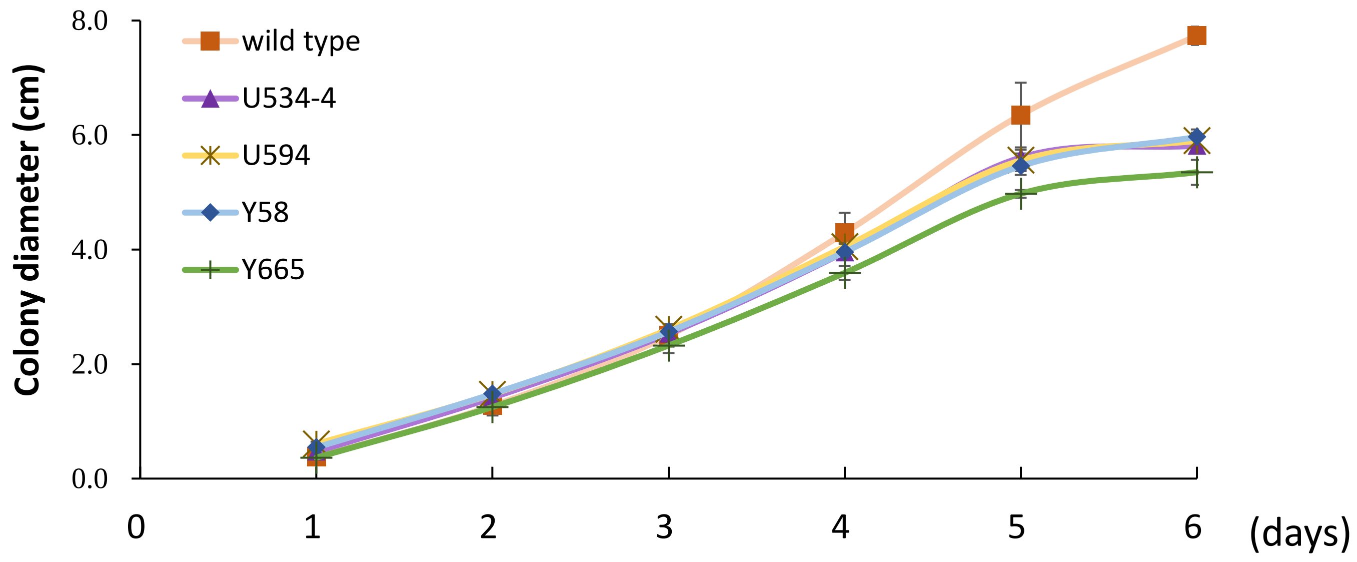

(B)

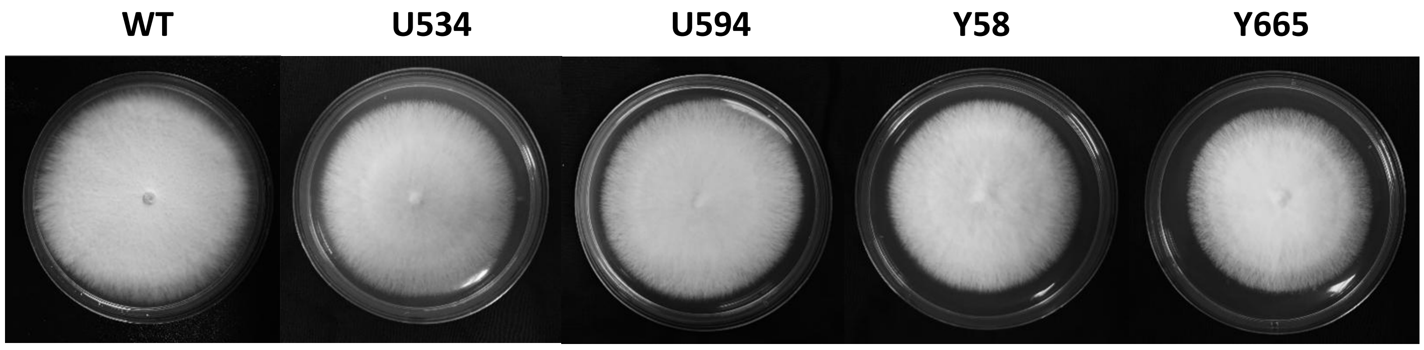

Figure S10. Growth and colony morphology of *Glmca1* (Y58 and Y665) and *URA3* (U534 and U594) silencing strains. Mycelial agar plugs were cultured on PDA for 6 days. (A) Colony diameters were measured and recorded daily. (B) Colony morphology was documented by photography on day 6. For each mutant, three biological replicates (n = 3) were used to calculate mean colony diameter and standard deviation. WT, wild-type

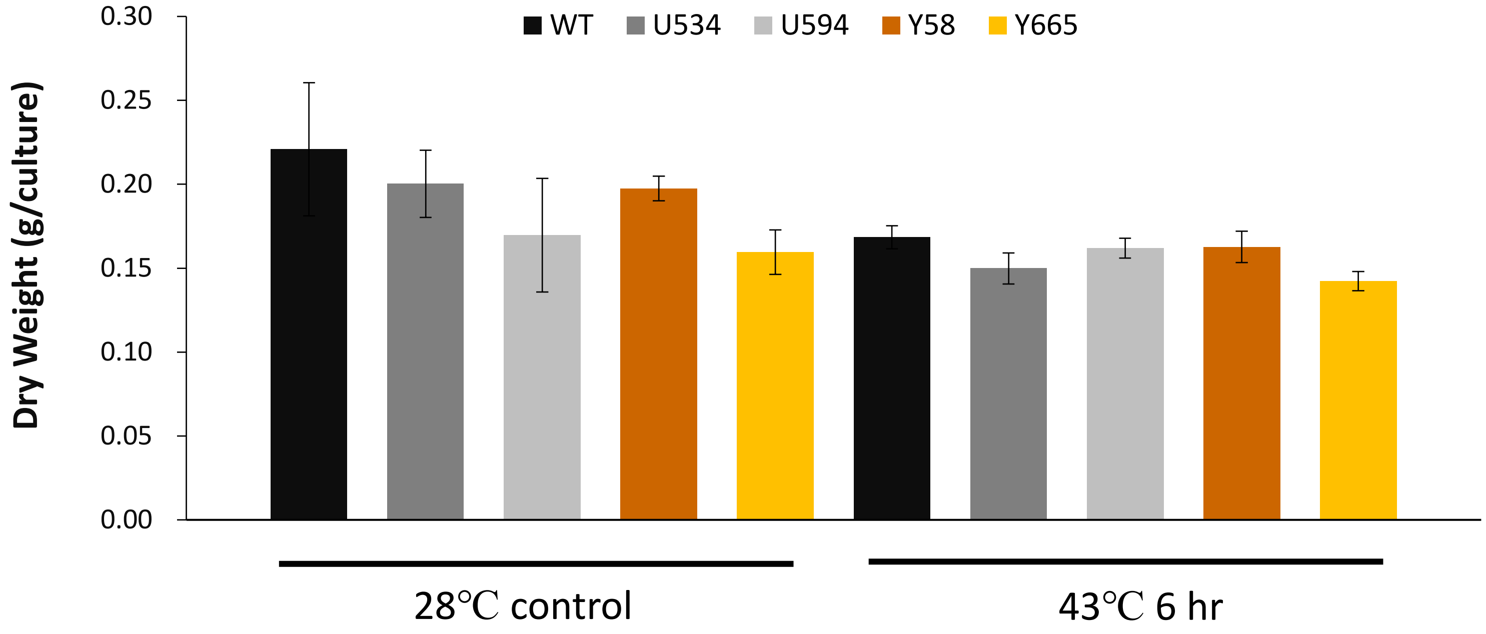

Figure S11. Biomass of the wild-type (WT), *URA3* silencing strains (U534 and U594), and *Glmca1* silencing strains (Y58 and Y665) after heat stress at 43 ˚C for 6 hours.
